# Differential Regulation of Duplicate Light-Dependent Protochlorophyllide Oxidoreductases in the Diatom *Phaeodactylum tricornutum*

**DOI:** 10.1371/journal.pone.0158614

**Published:** 2016-07-01

**Authors:** Heather M. Hunsperger, Christopher J. Ford, James S. Miller, Rose Ann Cattolico

**Affiliations:** Department of Biology, University of Washington, Seattle, Washington, United States of America; Stazione Zoologica Anton Dohrn, Naples, ITALY

## Abstract

**Background:**

Diatoms (Bacilliariophyceae) encode two light-dependent protochlorophyllide oxidoreductases (POR1 and POR2) that catalyze the penultimate step of chlorophyll biosynthesis in the light. Algae live in dynamic environments whose changing light levels induce photoacclimative metabolic shifts, including altered cellular chlorophyll levels. We hypothesized that the two POR proteins may be differentially adaptive under varying light conditions. Using the diatom *Phaeodactylum tricornutum* as a test system, differences in POR protein abundance and *por* gene expression were examined when this organism was grown on an alternating light:dark cycles at different irradiances; exposed to continuous light; and challenged by a significant decrease in light availability.

**Results:**

For cultures maintained on a 12h light: 12h dark photoperiod at 200μE m^−2^ s^−1^ (_200_L/D), both *por* genes were up-regulated during the light and down-regulated in the dark, though *por*1 transcript abundance rose and fell earlier than that of *por*2. Little concordance occurred between *por*1 mRNA and POR1 protein abundance. In contrast, *por*2 mRNA and POR2 protein abundances followed similar diurnal patterns. When _200_L/D *P*. *tricornutum* cultures were transferred to continuous light (_200_L/L), the diurnal regulatory pattern of *por*1 mRNA abundance but not of *por*2 was disrupted, and POR1 but not POR2 protein abundance dropped steeply. Under 1200μE m^−2^ s^−1^ (_1200_L/D), both *por*1 mRNA and POR1 protein abundance displayed diurnal oscillations. A compromised diel *por*2 mRNA response under _1200_L/D did not impact the oscillation in POR2 abundance. When cells grown at _1200_L/D were then shifted to 50μE m^−2^ s^−1^ (_50_L/D), *por*1 and *por*2 mRNA levels decreased swiftly but briefly upon light reduction. Thereafter, POR1 but not POR2 protein levels rose significantly in response to this light stepdown.

**Conclusion:**

Given the sensitivity of diatom *por*1/POR1 to real-time light cues and adherence of *por*2/POR2 regulation to the diurnal cycle, we suggest that POR1 supports photoacclimation, whereas POR2 is the workhorse for daily chlorophyll synthesis.

## Introduction

### POR and LIPOR function

**Fig 1:**
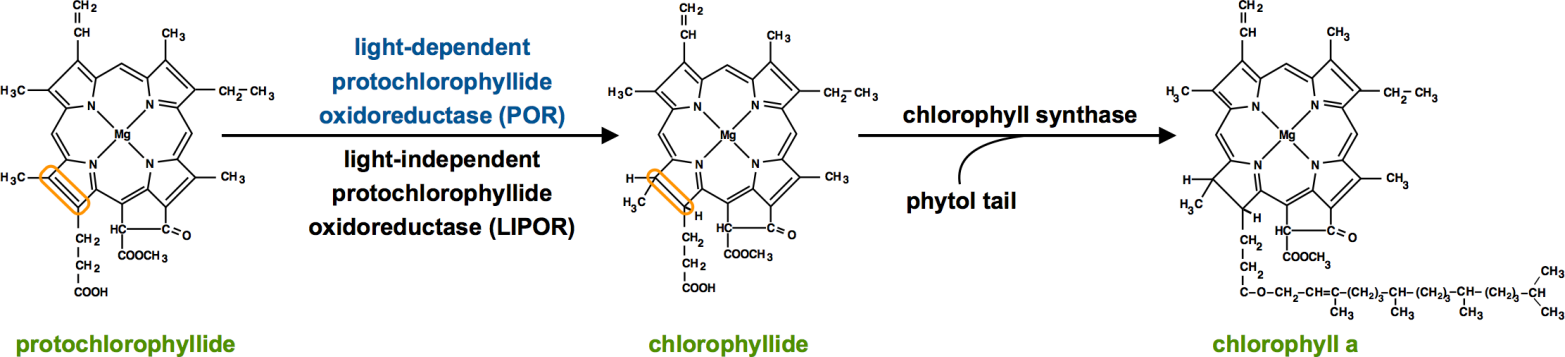
The final steps of chlorophyll *a* synthesis. The transformation of the chlorophyll pigment precursor protochlorophyllide into chlorophyllide can be catalyzed by either the light-dependent (POR) or light-independent (LIPOR) protochlorophyllide oxidoreductases. Upon the addition of a phytol tail to chlorophyllide by chlorophyll synthase, chorophyll *a* synthesis is completed.

Two functionally equivalent but non-homologous enzymes catalyze the penultimate step of chlorophyll synthesis: the light-dependent (POR) and light-independent (LIPOR) protochlorophyllide oxidoreductases [1,2]. Both proteins reduce the C17 = C18 double bond of the chlorophyll precursor protochlorophyllide (Pchlide) to form chlorophyllide (Chlide). The addition of a phytol tail to chlorophyllide by chlorophyll synthetase results in a mature chlorophyll *a* molecule (Fig 1). The long held assumption that most algae use both POR and LIPOR to generate chlorophyll was recently revised by the discovery that many algal taxa (e.g., many stramenopile, haptophyte, chlorarachniophyte and euglenid representatives) lack LIPOR and instead maintain multiple POR isoenzymes [3]. A similar pattern of LIPOR gene loss and POR expansion has also been documented to occur in diverse angiosperms such as *Arabidopsis*, barley, tobacco, tomato, corn, rice, as well as gymnosperms within the genus *Pinus* (reviewed in [4]). Interestingly, the expanded *por* gene sets of diverse angiosperms and algae have arisen from many unique duplication events specific to particular taxa, rather than through a shared ancient duplication event [3,4]. Whereas *por* gene regulatory schemes have been studied in many angiosperms (reviewed in [4]), little data exist for the recently documented *por* gene sets in algae. Our study addresses this paucity of knowledge by analyzing the regulation of *por* genes in the diatom *Phaeodactylum tricornutum* (Stramenopila: Bacilliariophyceae).

### POR evolution in eukaryotes

The origin of the first *por* gene and all other photosynthesis-related genes in algae can be traced to the endosymbiotic entrainment of a proto-cyanobacterium in a eukaryotic host cell [5]. The rhodophytic (red) and chlorophytic (green) algal lineages diverged ~1500 million years ago from this eukaryote-prokaryote chimera [6–8]. Whereas modern green algae and land plants emerged directly from the green algal lineage, other ‘green-lineage’ algal taxa have been established via unique secondary endosymbioses of green algae (and therefore green algal genes) that were incorporated into previously non-photosynthetic eukaryotic taxa (e.g., chlorarachniophyte and euglenid algae) [9,10]. Similarly, secondary or potentially higher order endosymbioses [11,12] involving the assimilation of red algae as chloroplasts have produced additional ‘red-lineage’ algae (e.g., the stramenopiles, haptophytes, cryptophytes and dinoflagellates). Phylogenetic analyses suggest that the duplication events leading to two *por* genes in both euglenids and chlorarachniophytes occurred after each of these algal lineages were established [3]. Uniquely, nearly all stramenopiles (including diatoms) and haptophytes appear to have lost their native red algal *por* genes. Instead, both of these algal lineages share duplicates of a *por* gene obtained via horizontal gene transfer from the ancestral prasinophyte lineage of green algae. Phylogenetic analyses suggest that the stramenopiles first incorporated and duplicated the prasinophytic *por* gene, and that this dual gene set was then transferred to haptophytes in a separate horizontal or potentially endosymbiotic gene transfer event (see [3] for further discussion).

### POR specialization

The maintenance of redundant gene sets for extended evolutionary time periods is ascribed to divergences in biochemistry or regulation of their resultant product(s) that offer adaptive advantages [13]. Most simply, gene dosage can increase when additional gene duplicates are conserved. Alternatively, mutations of the coding or regulatory sequences can divide enzymatic responsibility between gene duplicates (sub-functionalization) or enable the rise of novel functions (neo-functionalization) [14,15]. Because the *por* gene families of stramenopiles/haptophytes, chlorarachniophytes, euglenids, and land plants were individually established via independent gene duplication events, each of these expanded *por* gene families evolved separately [3]. Because each of these taxa possesses vastly different evolutionary histories, nuclear gene complements, regulatory networks, physiologies and ecologies, one may posit that each POR isoenzyme fulfills different needs for each organism. Given the universality of *por* gene duplication across evolutionarily distant lineages, one must also consider the possibility that convergent evolution in POR enzyme regulation and/or function in response to similar environmental stimuli might occur.

A particularly well-studied *por* gene expansion is that observed in the land plant *Arabidopsis thaliana*. This organism maintains three nuclear-encoded *por* genes (*por*A, *por*B, *por*C) encoding unique POR enzymes (PORA, PORB, PORC) that each fulfills a specific role upon transit to the chloroplast thylakoid membrane. *A*. *thaliana por*A is highly transcribed and translated in dark-adapted seedlings, poising tissues for rapid greening upon exposure to light [16]. The gene *por*B is under circadian regulation, supporting daily chlorophyll synthesis [17]. The third gene, *por*C, is up-regulated in response to increasing light intensities and is postulated to enable elevated rates of chlorophyll synthesis under high light [17]. Similar to *A*. *thaliana*, the multiple *por* genes of other angiosperms as well as those of gymnosperms display unique regulatory schemes for each gene copy. Some species appear to share similar *por* gene regulatory programs even though their *por* genes arose from unique duplication events during evolution [4]. For example, *Hordeum vulgare* (barley) also has a *por* gene specialized to seedling greening and another for daily chlorophyll synthesis although these *por* gene duplicates arose independently from those of *A*. *thaliana* [18].

Whether the regulatory schemes of algal *por* genes have converged with those of vascular plants is unknown. Diurnal fluctuations in light availability present a powerful and nearly universal environmental signal for metabolic entrainment [19]. When the diatom alga *P*. *tricornutum* was exposed to a light/dark photoperiod, cellular chlorophyll content increased in the light and decreased in the dark [20]. Upon continuous illumination, cellular chlorophyll content rose in the subjective day, decreased in the subjective night, and increased considerably just prior to subjective dawn. Though evidence of circadian regulation of chlorophyll synthesis in *P*. *tricornutum* is indirect, one may posit that at least one of *P*. *tricornutum*’s *por* genes is regulated similarly to the *Arabidopsis por*B gene.

However, specialization unique to algal *por* gene duplicates must also be considered given that physiological responses can differ between phytoplankton and land plants. For example, whereas land plants generally increase chlorophyll levels under high light conditions [21], both cyanobacteria and eukaryotic algae increase chlorophyll concentrations under low light intensities as part of longer-term (hours to days) photoacclimative responses [22–24]. Modifications in enzyme regulation or catalytic efficiency would additionally allow cells to respond to physiological stresses unique to phytoplankton, such as light fluctuations caused by vertical displacement within the water column, self-shading during a bloom, or the presence of detritus.

### Diatom POR regulation

The recent sequencing of genomes and transcriptomes from a broad representation of diatoms revealed that at least 22 of 24 sampled species possess *por* gene duplicates [3]. Importantly, all six fully sequenced diatom chloroplast genomes lack LIPOR genes [3], underscoring the dependence of these algae on light for POR protein function in chlorophyll production. Using the model diatom *P*. *tricornutum* [25–27], we assessed how these algae use their dual POR enzymes when exposed to changing light regimes. *P*. *tricornutum por*1 and *por*2 mRNA as well as POR1 and POR2 protein abundances were analyzed both when cultures were maintained on alternating light:dark photoperiods and when cultures were subject to significant shifts in light availability (i.e., exposure to constant light or a profound reduction in light quantity). By tracking responses to these changing light programs over multiple days, normal diurnal activities as well as responses associated with photoacclimation were monitored. Results show each *P*. *tricornutum por* gene to be uniquely regulated, and suggest that that diatom *por*1/POR1 may play a role in photoacclimation to low light whereas *por*2 transcription and POR2 protein abundance are under diurnal regulation.

## Results

### Characterization of *P*. *tricornutum* PORs

**Fig 2:**
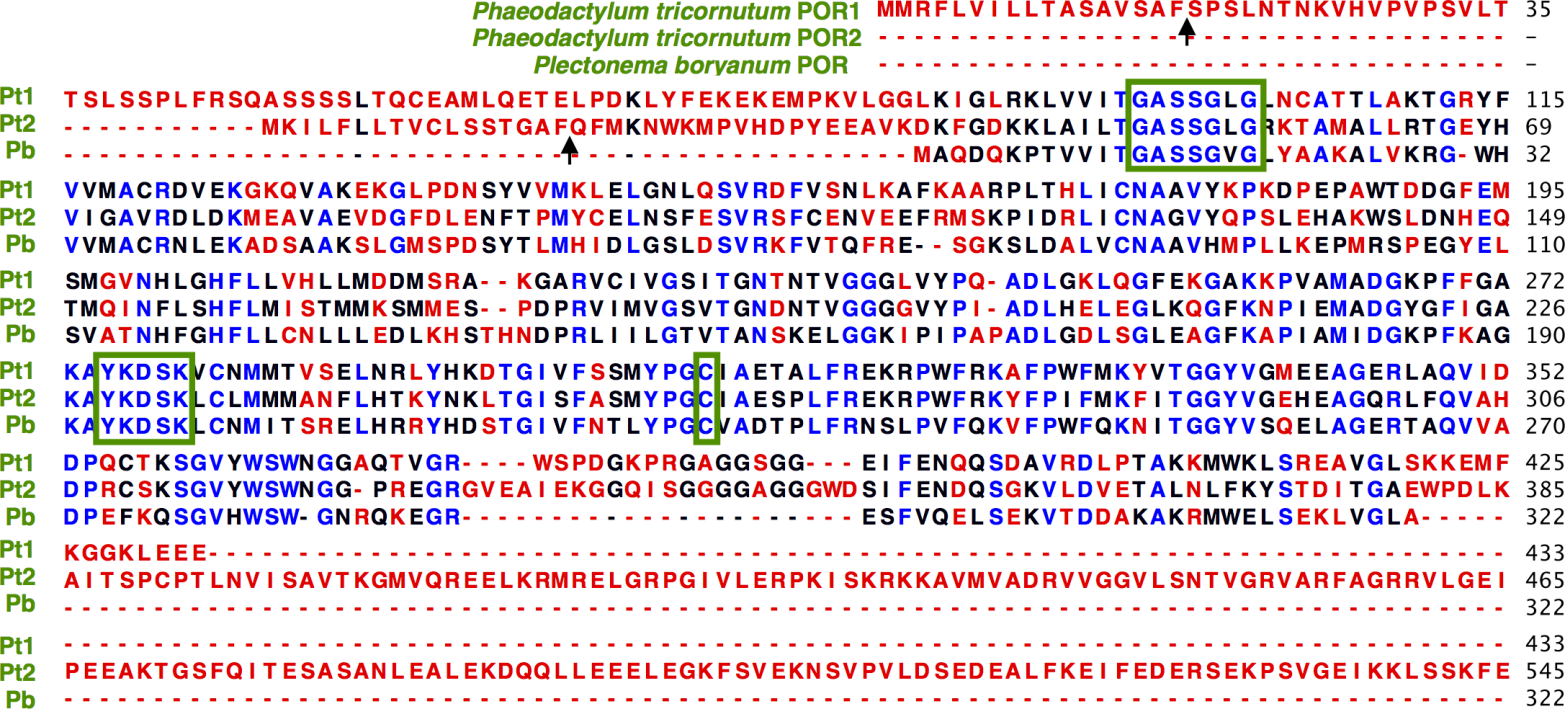
Multiple sequence alignment of diatom *P*. *tricornutum* and cyanobacterium *Plectonema boryanum* POR proteins. Color indicates amino acid sequence conservation across the three PORs: highly conserved (blue), semi-conserved (black), and poorly conserved (red). Green boxes indicate the N-terminal Rossman fold (GxxxGxG) for NADPH-binding [31], YxxxK active-site whose tryptophan (Y) donates a protein to Pchlide during catalysis [32–35], as well as the universally conserved cysteine (C) residue indispensable to proper POR enzyme conformation [36]. Arrows indicate signal peptide cleavage sites. The C-terminal extension of POR2 may not be present in the mature protein (see Discussion).

The hypothetical sizes of the POR1 and POR2 proteins are ~47kD and ~61kD, respectively, including putative bipartite signal/transit peptides. Using the HECTAR algorithm [28] to identify the bipartite targeting peptide of stramenopile chloroplast-targeted proteins, POR1 was predicted to be chloroplast-localized with high confidence, demonstrating both canonical signal and transit peptides (Fig 2) needed for transporting proteins across the four membranes that enclose stramenopile plastids. Although only the signal peptide was identified for POR2, alternative methods for protein movement (e.g., vesicular transport) within *P*. *tricornutum* plastids have been reported [29,30].

**Fig 3:**
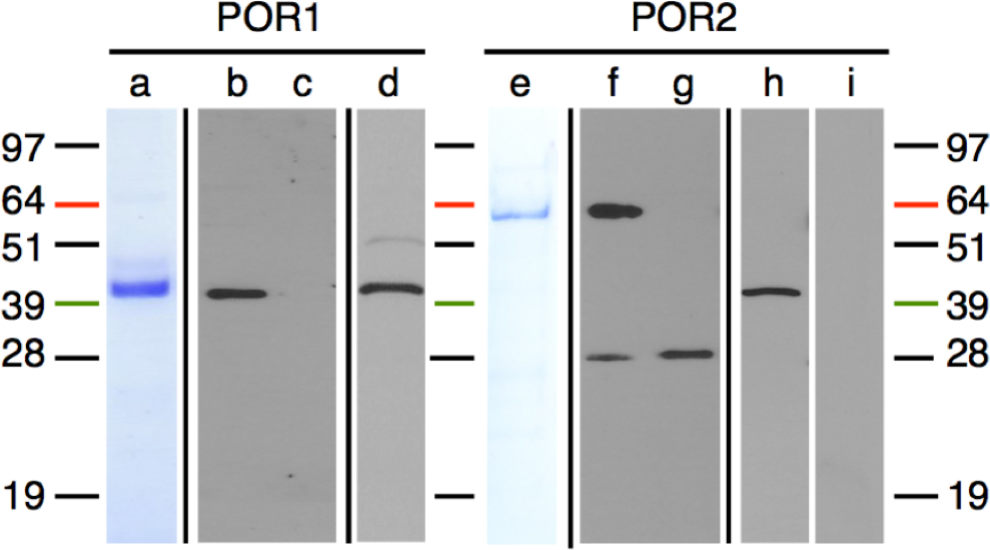
POR1 and anti-*P*. *tricornutum* POR2 antibodies. (a) Heterologously expressed and affinity-purified POR1 protein for antibody production. The anti-POR1 antibody recognized (b) affinity-purified POR1 protein, but not (c) affinity-purified POR2 protein. (d) Reactivity of the affinity-purified POR1 antibody against *P*. *tricornutum* protein extracts. (e) Heterologously expressed and affinity-purified POR2 protein for antibody production. The anti-POR2 antibody recognized (f) affinity-purified POR2 protein, but not (g) affinity-purified POR1 protein. The additional band in (f) and (g) represents an *E*. *coli* protein that co-purified from (a) and (e) but is not found in *P*. *tricornutum* [see (h)]. (h) Reactivity of the anti-POR2 antibody to *P*. *tricornutum* extracts. (i) Reactivity of the anti-POR2 antibody to *P*. *tricornutum* extracts after antibody incubation with affinity-purified POR2 protein. Black bars separate images from different gels/blots.

Both *por*1 and *por*2 cDNAs obtained by 3’ RACE matched protein translations predicted in the genome annotations [37], confirming the hypothetical protein sizes of POR1 and POR2. To generate antibodies to POR proteins, cDNAs were cloned into a pET-15-HE vector and expressed in bacteria (see Methods). The resultant, affinity purified anti-POR1 antibody detected heterologously expressed POR1 but not POR2 proteins, and cross-reacted with a 42.5kD band of the approximately expected size for POR1 (Fig 3B–3D). The anti-POR2 antibody cross-reacted with heterologously expressed POR2 proteins but not POR1 proteins, and detected a single protein band in *P*. *tricornutum* extracts (Fig 3F–3H). The band detected by the anti-POR2 antibody was 44kD, which is much smaller than the expected ~59kD protein based on 3’ RACE of the mRNA transcript. However, the 44kD band was eliminated by first ‘blocking’ the anti-POR2 antibody with affinity-purified POR2 protein (Fig 3E), demonstrating that this band indeed represents a POR2 protein. These results show specificity of each *P*. *tricornutum* anti-POR antibody to its particular isomer.

### Culture specifications for light response studies

**Fig 4:**
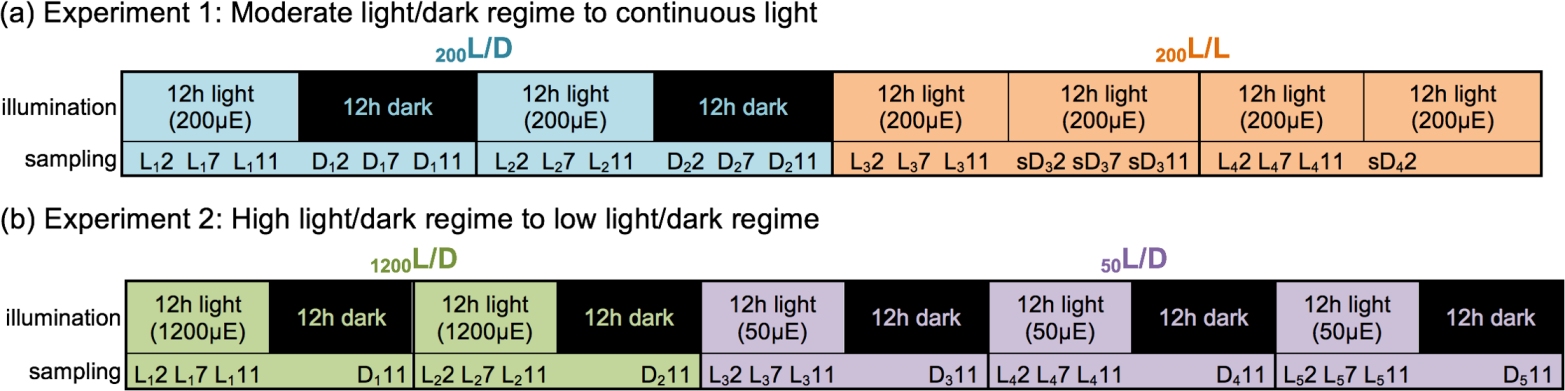
Experimental series. In Experiment 1 (a), *P*. *tricornutum* cells acclimated to a 12h light: 12h dark (L/D) photoperiod at 200μE m^−2^ s^−1^ (_200_L/D) were transferred to continuous light (L/L) of the same intensity (_200_L/L). In Experiment 2 (b), culture acclimated to a 12h light: 12h dark photoperiod at 1200μE m^−2^ s^−1^ (_1200_L/D) was shifted to 50μE m^−2^ s^−1^ on the same photoperiod (_50_L/D). In Experiment 1, samples were collected at hours 2, 7, and 11 of each 12h phase (thus in each light phase at L2, L7, and L11; in each dark phase at D2, D7, and D11; and in each subjective dark phase at sD2, sD7, and sD11). In Experiment 2, samples were collected at hours L2, L7, L11, and D11. The subscripted number after L or D indicates the 24h photoperiod during which that sample was collected (e.g., D_2_7 is the seventh hour of the dark phase pertaining to the second photoperiod of that experiment).

The regulatory responses of *por*1/POR1 and *por*2/POR2 to shifts in light regime were explored in the two experiments outlined in Fig 4. The first study (Fig 4A) followed *P*. *tricornutum* cells habituated to a light:dark photoperiod as they were shifted to continuous light. The second study (Fig 4B) monitored cells grown under high light conditions as they acclimated to low light conditions. *P*. *tricornutum* cells were grown as semi-continuous cultures in a 15L photobioreactor containing 12L of medium to provide a uniform culture source (S1 Fig). To ensure that shifts in light intensity represented the sole variable under investigation, physiological parameters were carefully regulated. To eliminate issues of self-shading or nutrient depletion over the course of extended sampling periods, photobioreactor contents were diluted each 24h period at the onset of the light (L0) with fresh F/2 medium. Approximately 20–30% of the culture was harvested every 24h (15% during the light period).

*P*. *tricornutum* growth responses in the 15L photobioreactor are shown in S2 Fig for the semi-continuous experimental cultures as well as for batch cultures grown either under a 12h light: 12h dark photoperiod (L/D) or under constant illumination (L/L) at 50μE m^−2^ s^−1^ (_50_L/D), 200μE m^−2^ s^−1^ (_200_L/D and _200_L/L) and 1200μE m^−2^ s^−1^ (_1200_L/D). Cell growth in the semi-continuous cultures, regardless of light regime, paralleled those obtained in batch cultures (S2 Fig). The moderate light intensity of 200μE m^−2^ s^−1^ (_200_L/D) was chosen to serve as a reference for the high and low light studies described below.

### Experiment 1: Moderate light/dark regime

#### Cell growth responses and chlorophyll production

**Fig 5:**
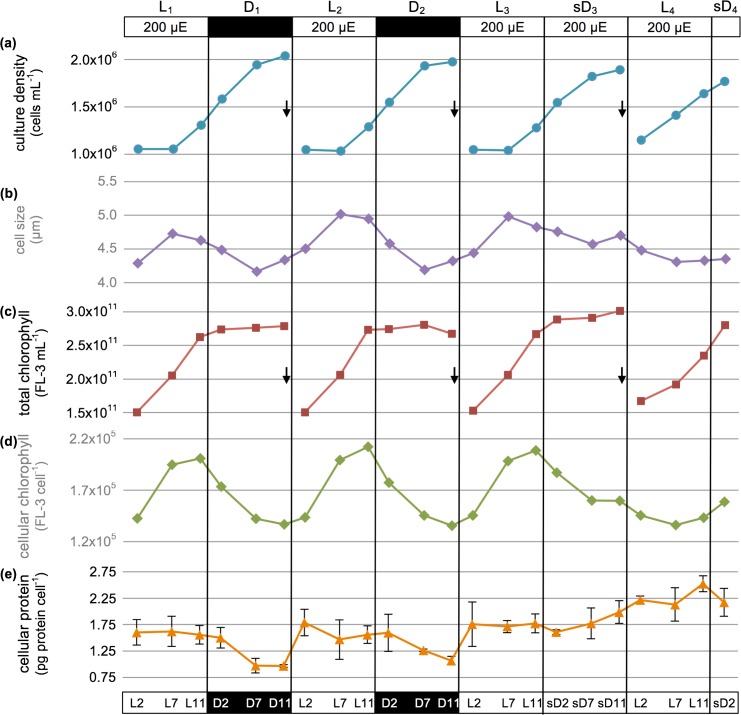
Growth and physiology under moderate diurnal light (_200_L/D) followed by transition to constant illumination (_200_L/L). Culture acclimated to _200_L/D was sampled for two days prior to and 1.6 days after a transition to _200_L/L. Arrows indicate daily dilution with fresh medium at L0 to ~1.0 x10^6^ cells/mL. (a) Culture density (cells/mL); (b) size (μm); (c) total chlorophyll (FL-3 fluorescence/mL); (d) cellular chlorophyll (FL-3 fluorescence/cell); and (e) cellular protein (pg/cell; error bars show standard deviation). The culture was acclimated for 4.2 generations (4 days) under _200_L/D before sampling began.

To determine the effect of a moderate light regime on *P*. *tricornutum por* transcript and POR protein production, cells were sampled over a two day period when grown under a 12h light: 12h dark light/dark cycle at 200μE m^−2^ s^−1^ (_200_L_1_/D_1_ and _200_L_2_/D_2_; Fig 5). Cultures were partially synchronized by this light/dark regime, dividing approximately once daily. Division for both days initiated by the 7^th^ hour in the light (i.e., L_1_7 and L_2_7) and ceased by the 7^th^ hour in the dark (i.e., D_1_7 and D_2_7; Fig 5A), in a pattern that repeated itself in both phase and amplitude. Average cell size was maximal in the middle of the light period (~4.9μm) and minimal at the termination of cell division (~4.2μm; Fig 5B). *P*. *tricornutum* is only lightly silicified and thus size is not reduced with each division cycle as in other diatoms [38]. Changes in both total and cellular chlorophyll *a* abundance (hereafter chlorophyll, as measured by cellular fluorescence; Fig 5C and 5D and S3 Fig) showed a highly reproducible phase, period, and amplitude when monitored over the two-day sampling period. Total chlorophyll abundance increased linearly over the course of each day (e.g., _200_L_1_2 to _200_L_1_11). Mean chlorophyll abundance per cell, however, slowed upon the onset of cell division (e.g., _200_L_1_7 to _200_L_1_11) and thereafter declined until the termination of the dark cycle (e.g., _200_D_1_12). Average cellular protein levels for both L_1_/D_1_ and L_2_/D_2_ cycles remained high in the light and declined to lowest values by the end of the dark portion of the cycle, coincident with the termination of cell division (Fig 5E). Taken together, *P*. *tricornutum* cellular division, as well as chlorophyll and protein abundance appeared to be highly consistent across photoperiods and tightly coupled to light cues.

#### Transcriptional regulation of *por*1 and *por*2

Given the potential redundancy of *por*1 and *por*2, it was of interest to know whether transcript abundance of these two genes differed as *P*. *tricornutum* progressed through the diurnal cycle. Four reference genes proposed for qPCR abundance normalization standards in *P*. *tricornutum* light/dark cycle studies [39] were evaluated for use in this analysis. Because analysis with BestKeeper software [40] showed low standard deviations and high coefficients of correlation for genes *cdkA* (cyclin-dependent kinase A) and *TBP* (TATA box binding protein), these two genes were used as reference genes in Experiment 1 (S4 Fig).

**Fig 6:**
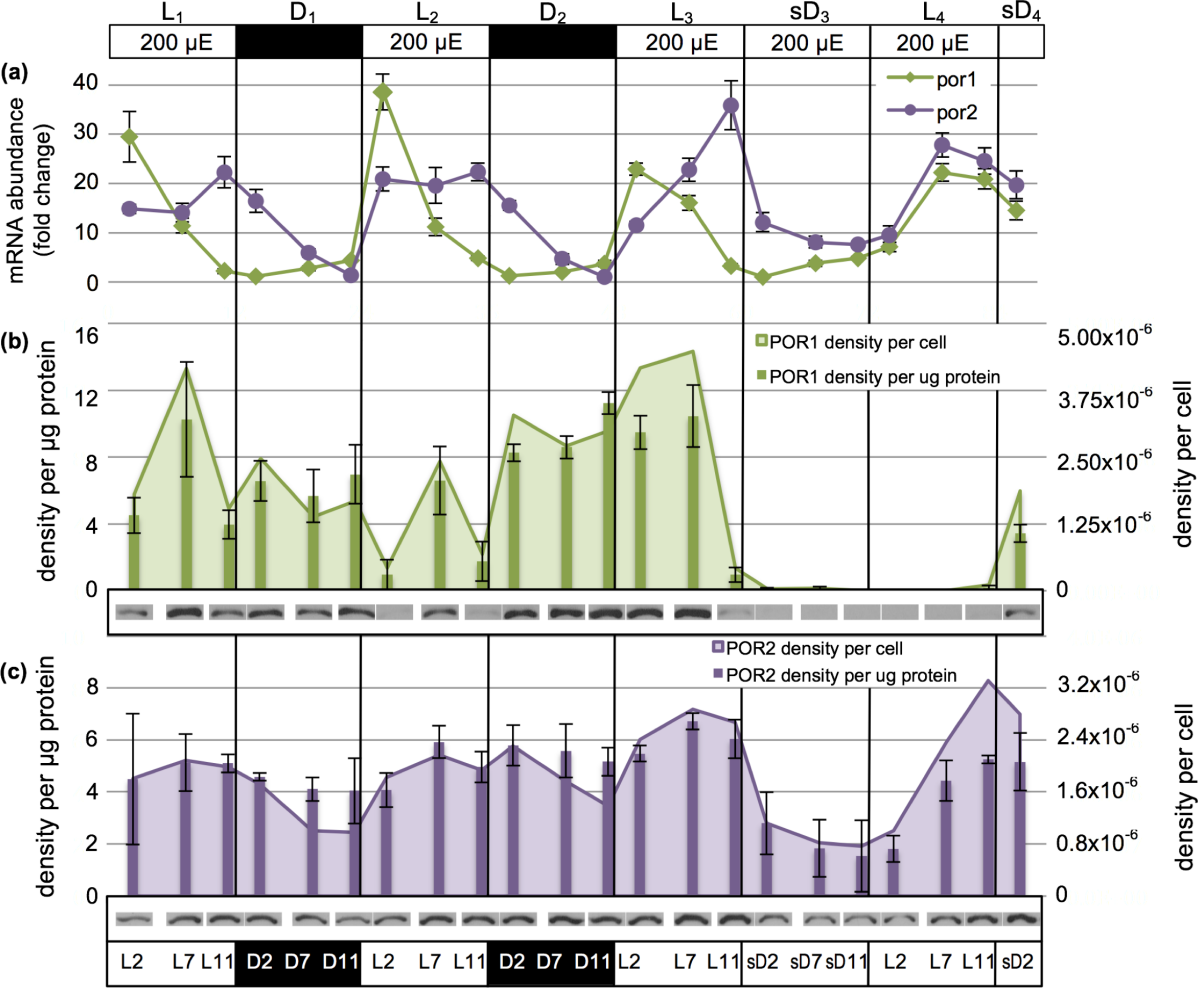
*Por* mRNA transcript and POR protein abundance under moderate diurnal light (_200_L/D) transitioning to constant illumination (_200_L/L). (a) Fold-change in *por*1 and *por*2 mRNA transcript abundance measured by RT-qPCR. (b) POR1 density per μg protein measured by Western blotting (bars; error bars show standard deviation) and normalized to cellular protein levels to attain POR1 density per cell (area chart). Representative blot data are shown below each sampled time point. (c) As in (b) but for POR2.

Messenger RNA abundance for *por*1 oscillated reproducibly over two 24h _200_L/D cycles (Fig 6A). A small increase in *por*1 product appeared at the end of the dark, ramped up to a high abundance early in the light period, followed by a marked decline by light hour 7 (L7). Though transcript abundance of *por*2 was also maximal in the light, this RNA peaked in abundance later in the light period and rapidly declined as cells transitioned into the dark period. Thus, *por*1 and *por*2 RNA transcripts were both up-regulated in the light and down-regulated in the dark, but displayed a phase shift relative to one another.

#### POR1 and POR2 enzyme accumulation

Although POR1 protein abundance fluctuated during both light (_200_L_1_, _200_L_2_ and _200_L_3_) and dark (_200_D_1_ and _200_D_2_) phases of growth, temporal reproducibility in the concentration of this enzyme was not observed (per cell or per μg protein; Fig 6B). In contrast, POR2 enzyme accumulation per μg protein remained fairly stable throughout the light/dark cycle. At the cellular level, however, a shallow oscillation was seen in response to light/dark cues; cellular POR2 levels increased modestly during _200_L_1_, _200_L_2_ and _200_L_3,_ then declined throughout _200_D_1_ and _200_D_2_, reaching a minimum at the end of the dark phase (Fig 6C). These cell-level oscillations likely reflect both POR protein anabolism/catabolism over the light:dark photoperiod as well as cellular protein reduction due to cell division (Fig 5A and 5E). Supporting the findings of Fig 6, all three light/dark regimes of Experiments 1 and 2 (_200_L/D, _1200_L/D, and _50_L/D; see Fig 4) showed a _200_L/D-like pattern for POR2 per μg protein as well as POR2 per cell. Light and dark interval POR2 abundances *per cell* were significantly different when analyzed for these three light/dark regimes (*p = 0*.*003*), whereas no difference was detected at the level of POR2 abundance *per μg protein* (*p = 0*.*250*). Incorporation of _200_L_3_ and _200_sD_3_ data (Experiment 1) into the analysis of *cellular* POR2 levels under _50_L/D, _200_L/D and _1200_L/D increased statistical significance of the diurnal oscillatory response (p = 0.00087).

Data show that there was no strong correspondence between *por*1 RNA abundance and POR1 protein levels over the diurnal cycle–suggesting that the regulation of POR1 protein level most likely occurs post-transcriptionally. A closer, though imperfect, correspondence between *por2* mRNA and cellular POR2 enzyme levels was seen. Most intriguing, given the light requirement of the POR enzyme for catalysis, was the retention of high levels of POR1 and POR2 enzymes in the dark phases of the diurnal cycle when compared to the light phases.

### Experiment 1: Photoacclimation from moderate light/dark regime to constant illumination

#### Cell growth responses and chlorophyll production

To determine the impact of eliminating the dark cue on POR enzyme biogenesis, the *P*. *tricornutum* culture previously maintained for two 12h light: 12h dark cycles (L_1_/D_1_ and L_2_/D_2_) was shifted to a continuous light regime while maintaining the same light intensity (200μE m^−2^ s^−1^). In the first 24h on this new program, cell division proceeded from _200_L_3_7 to subjective night _200_sD_3_7 as expected under a normal light/dark cycle, but achieved only a 0.89 cell divisions per day rather than the 1.0 per day observed in previous _200_L/_200_D cycles (Fig 5A, right half). Additionally, the continued lack of a dark cue most likely resulted in the early onset of cell division during the ensuing _200_L_4_/sD_4_ cycle. Division began by _200_L_4_0, and appeared to persist through the termination of the experiment at _200_sD_4_2.

The shift in the timing of cell division for cultures grown in continuous light also impacted cell size (Fig 5B, right half). Although a maximum average cell size of 5.0μm was attained by _200_L_3_7 in the first continuous light cycle, average cell size only declined to 4.6μm by _200_sD_3_7 rather than the expected 4.2μm minimum. Subsequently, cell size no longer achieved the _200_L/D maximum, but continued to slowly and linearly decrease until the cells achieved a size (4.3μm) similar to that normally observed at _200_L/D post-division (4.2μm). These data plus the linear increase in protein per cell beginning at _200_L_4_2 (Fig 5E) suggest that the culture began to lose a phased division response and started to divide continuously.

Under the continuous light regime, total chlorophyll abundance increased in the _200_L_3_ culture similarly to that observed in _200_L_1_ and _200_L_2_ (Fig 5C). Chlorophyll accumulation continued in the subjective dark of _200_sD_3_, reaching fluorescence levels approximately 10% higher than previous dark periods, reflecting the increase in cell size (Fig 5B). Chlorophyll abundance per cell at _200_sD_3_7 was equal to that seen in _200_D_1_7 and _200_D_2_7 (Fig 5D). However, from _200_sD_3_7 to _200_sD_3_11, average chlorophyll per cell did not decline as rapidly as anticipated for a normal dark period, nor increased from _200_L_4_2 to _200_L_4_11 as expected for a normal light period. Overall chlorophyll decline per cell most likely was due to the early cell division that took place in the L_4_ cycle (Fig 5A) combined with the decrease in total chlorophyll abundance (Fig 5C).

#### Transcriptional regulation of *por*1 and *por*2

Despite a continuous light regime, *por*1 transcript levels reached an expected low at the onset of the subjective dark phase at sD_3_ and then slowly increased in abundance in sD_4_, as expected on an _200_L/D cycle (Fig 6A, right half). Lacking the dark reset however, *por*1 mRNA abundance was not highest at the onset of the light phase as found under the _200_L/D regime. Instead, *por*1 transcript increase was delayed until the middle of L_4_, indicating that a phase shift had occurred. Transcript accumulation for *por*2 appeared to maintain a normal oscillation whether the cultures were subject to a _200_L/D or _200_L/L light program, a result encouraging future studies to determine whether the gene is under circadian regulation. Interestingly, *por2* transcript abundance remained slightly elevated during the _200_sD_3_ when compared to levels observed during a true dark period (e.g. _200_D_1_ and _200_D_2_).

#### POR1 and POR2 enzyme accumulation

Despite the transition from an L/D regime to continuous illumination, POR1 proteins accumulated as expected during L_3_ (Fig 6B). Thereafter, the concentration of this protein began to drop precipitously after L_3_7 and was only marginally detectable for almost 24h when, at L_4_11, a slight increase in enzyme signal was seen. This signal was further augmented in sD_4_, suggesting a re-establishment of protein complement was potentially occurring. POR2 protein maintained the shallow oscillatory response in abundance per cell that peaked at approximately L7 for the _200_L/D regime (Fig 6C). However, POR2 abundance (both per cell and per μg protein) was much lower by sD_3_2 than at D_1_2 and D_2_2. Furthermore, POR2 abundance per μg protein remained at approximately half of the levels seen during a normal _200_L/D cycle through L_4_2. In the next expected light period, POR2 levels returned to typical _200_L/D levels.

### Experiment 2: High light/dark regime

#### Cell growth responses and chlorophyll production in high light cultures

**Fig 7:**
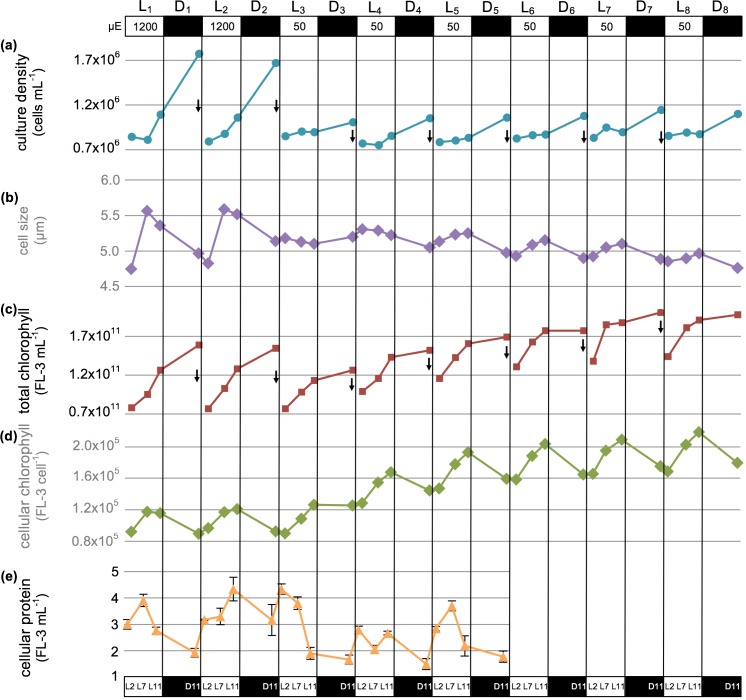
Growth and physiology under high-diurnal light (_1200_L/D) and transition to low-diurnal light (_50_L/D). Culture acclimated to _1200_L/D were sampled two days prior to and three days after a transition to _50_L/D, and then monitored for an additional three days. Arrows indicate daily dilution with fresh medium at L0 to ~0.8 x10^7^ cells/mL. (a) Culture density (cells/mL); (b) size (μm); (c) total chlorophyll (FL-3 fluorescence/mL); (d) cellular chlorophyll (FL-3 fluorescence/cell); and (e) cellular protein (pg/cell; error bars show standard deviation). The culture was acclimated for 8.5 generations (5 days) under _1200_L/D before beginning sampling.

To determine whether the regulation of *por*1/POR1 and *por*2/POR2 differ as *P*. *tricornutum* cells adjust to a light step-down, cells were sampled as they transitioned from a 1200μE m^-2^ s^-1^ L/D cycle (_1200_L/D) to a 50μE m^−2^ s^−1^ L/D cycle (_50_L/D) (Fig 7). The *P*. *tricornutum* population acclimated to _1200_L/D grew slightly faster (1.2 divisions per day) than the control culture grown at _200_L/D (1.0 division per day; Fig 5A) (Fig 7A). In the middle of the light period (L7, before cell division), the mean cell fluorescence of *P*. *tricornutum* grown at _1200_L/D was on average 40% lower than that seen when the diatom culture was maintained at _200_L/D. Thus, the high light culture displayed the expected increase in cell division rates and decrease in cellular chlorophyll levels [20,23]. Although chlorophyll levels were reduced during the two _1200_L/D cycles, the amount of chlorophyll per cell and protein per cell present displayed a similar period and phase in product abundance to that observed for the control _200_L/D grown cultures.

#### Transcriptional regulation of *por*1 and *por*2 and POR1 and POR2 enzyme accumulation

Because genes *cdkA* and *TBP* continued to show low standard deviations and high coefficients of correlation in BestKeeper ([40]; S4 Fig), they also served as reference genes for Experiment 2.

**Fig 8:**
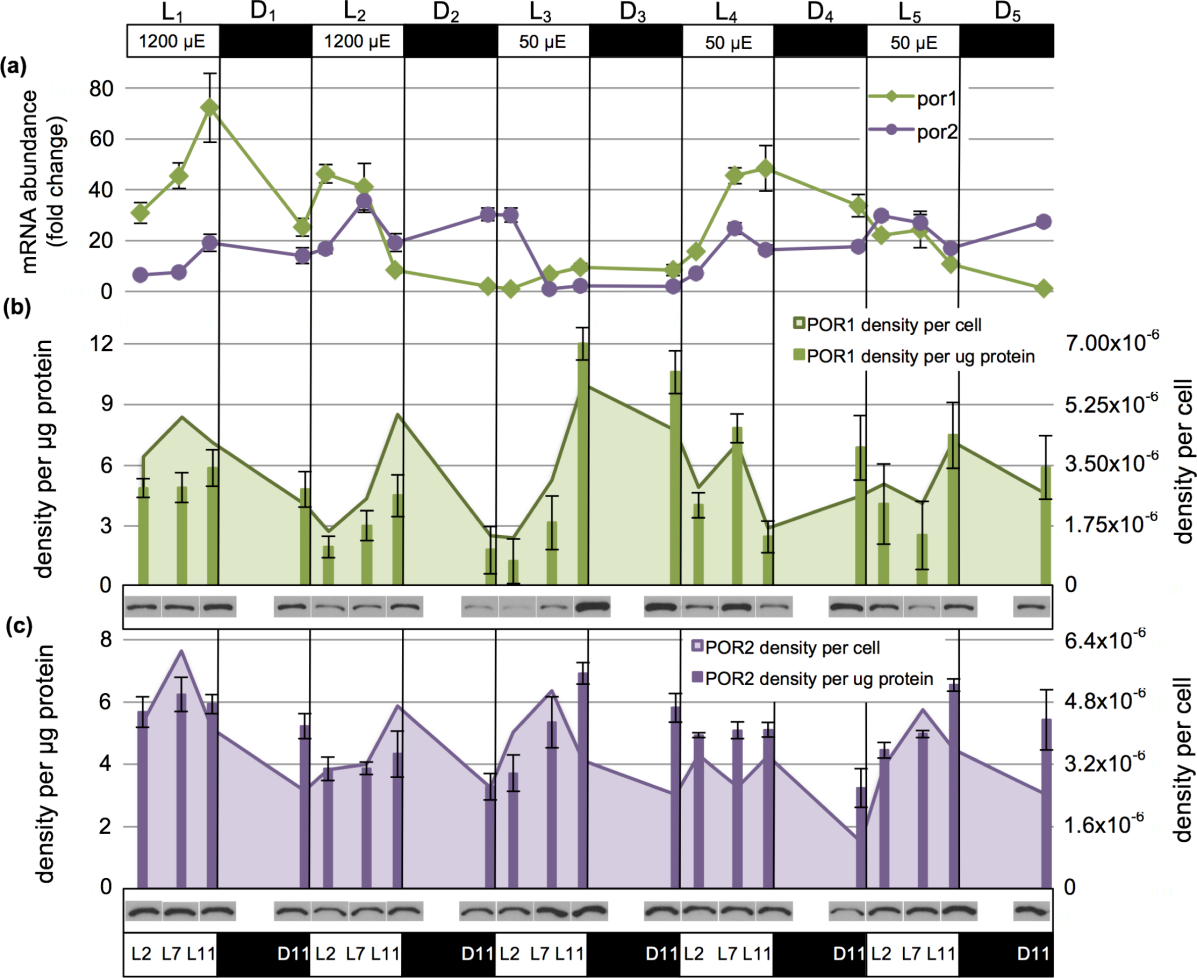
*Por* mRNA transcript and POR protein abundance under high-diurnal light (_1200_L/D) and transition to low-diurnal light (_50_L/D). (a) Fold-change in *por*1 and *por*2 mRNA transcript abundance as measured by RT-qPCR. (b) POR1 density per μg protein as measured by Western blotting (bars; error bars show standard deviation) and normalized to cellular proteins levels to attain POR1 density per cell (area chart). Representative blot data is shown below each sampled time point. (c) As in (b) but for POR2.

The high light exposure of 1200μE m^−2^ s^−1^ appeared to impact both *por* mRNA as well as POR protein abundance patterns when observed over the L/D cycle. Although both *por*1 and *por*2 gene products were most abundant in the light, the reproducible amplitude and periodicity seen in the _200_L/D culture was lost (Fig 8A). In contrast to the differences observed in both POR1 and POR2 protein _200_L/D patterns, those observed for _1200_L/D showed a strong, tightly coordinated oscillation at the cellular level: high in the light and low in the dark (Fig 8B and 8C).

### Experiment 2: Photoacclimation from high light/dark regime to low light/dark regime

#### Cell growth responses and chlorophyll production upon transition to low light

Transition to the _50_L/D light regime caused an immediate decrease in the rate of cell division to 0.26 divisions per day on _50_L_3_/D_3_ (Fig 7A). Cell division rose slowly as cells acclimated to the new light condition, reaching 0.33 divisions per day by _50_L_5_/D_5_ and maintaining approximately 0.38 divisions per day on _50_L_8_/D_8_, the same cell division rate seen in _50_L/D acclimated batch cultures in exponential growth (S2 Fig).

Average cell size initially remained high under _50_L/D (~5.2μm; Fig 7B). Mean cell size then decreased linearly as the cellular division rate slowly increased, with daytime maximum cell size averages much smaller than those observed under _1200_L/D (~4.9μm on the sixth day at _50_L/D compared to ~5.6μm under _1200_L/D). Protein abundance retained a predictable though imperfect oscillatory pattern due to cell growth and division over the _50_L/D cycle.

The transition from _1200_L/D to _50_L/D initially led to decreased total chlorophyll levels at the end of the _50_L_3_/D_3_ cycle (Fig 7C). However, by the end of the second low light photoperiod (_50_L_4_/D_4_), total chlorophyll levels in the culture had rebounded to the levels observed at _1200_L/D and continued rising. Similarly, a slow increase in chlorophyll per cell began upon transfer to _50_L/D. By the end of six days at the new _50_L/D regime, mean fluorescence per cell at L7 (before cellular division in the _1200_L/D culture) was 173% higher than under the _1200_L/D photocycle. At the level of the individual *P*. *tricornutum* cell, cellular chlorophyll accumulation rates (measured from L2-L7 to exclude post-L7 cellular division under _1200_L/D) dropped dramatically on the first day at _50_L/D, recovered by the second day at low light, and increased thereafter to ~138% of the rate at _1200_L/D. Given the higher rate of cellular chlorophyll synthesis measured at _50_L/D, it appears that both increased chlorophyll production and decreased cellular division may contribute to the increasing cellular chlorophyll levels.

It is interesting to note that total chlorophyll levels in *P*. *tricornutum* increased during the dark period under the 1200μE m^-2^ s^-1^ but not during the 50μE m^-2^ s^-1^ or 200μE m^-2^ s^-1^ light:dark regimes. This observation is similar to a previous report [22] wherein the diatom *Skeletonema costatum* also appeared to produce chlorophyll in the dark when grown at 1200μE m^-2^ s^-1^ but not at 50μE m^-2^ s^-1^. Given that the chlorophyll measurements were fluorometric for both the *P*. *tricornutum* and *S*. *costatum* studies, and that the POR enzymatic reaction is expected to be light-dependent, one might conclude that results are artifactual to the measurement technique. Conversely, research confirming light-dependency of the POR enzyme has not been reported for any diatom.

#### Transcriptional regulation of *por*1 and *por*2

When subject to a light stepdown from _1200_L/D to _50_L/D, *por*1 mRNAs failed to accumulate normally during the first day under the new reduced-light regime (_50_L_3_/D_3_) whereas *por*2 mRNA was abundant at L_3_2 but declined precipitously by L_3_7 (Fig 8A). Subsequent to this 24-hour acclimation period, a burst in both *por*1 and *por*2 mRNA accumulation was observed. The high levels of *por*1 mRNA observed at _50_L_4_11 dropped steadily through the end of the experiment at _50_D_5_, suggesting that an over-compensation in transcription had occurred. In contrast, *por*2 mRNA levels appeared to form shallow and incomplete oscillations with peaks during the light period, a pattern similar to that seen for _1200_L/D.

#### POR1 and POR2 enzyme accumulation

Despite a sharp drop in both *por*1 and *por*2 mRNA levels during the _50_L_3_/D_3_ light exposure, protein quantification gives a completely different view of POR1 and POR2 regulation in cells adapting to a new light regime (Fig 8B and 8C). POR1 protein levels per μg of total protein peaked after transition to _50_L/D, with much greater abundance levels at _50_L_3_11 and _50_D_3_11 than in the two previous _1200_L/D cycles (*p<0*.*0001*). Thereafter, POR1 protein abundance decreased to a less-defined L/D pattern than that seen in the _1200_L/D regime. Despite robust POR1 abundance peaks during transition from the _1200_L/D to the _50_L/D light regime, POR1 levels were not significantly different when the three _50_L/D photoperiods were compared to the two _1200_L/D photoperiods (*p = 0*.*191*). These data show that most changes in POR protein levels occurred during the first 24-hour transition to the lower light intensity. In contrast to these observations for POR1, the accumulation of POR2 enzyme under _50_L/D appeared to be regulated in a similar manner as that observed for _1200_L/D. The light phase-dominant increase in cellular levels of POR2 protein seen in the two _1200_L/D cycles was repeated in all three _50_L/D cycles.

## Discussion

### POR protein structures

*P*. *tricornutum* POR1 and POR2 protein sequences show similar levels of sequence conservation to one another (65% biochemical similarity) as to conventional POR enzymes (e.g., 54–65% similarity to that of the cyanobacterium *Plectonema boryanum*) when the N-terminal signal and transit peptides of POR1 and POR2 and the long C-terminal tail of POR2 are excluded (Fig 2). For comparison, the three *A*. *thaliana* POR enzymes and two *H*. *vulgare* POR enzymes show 84–94% and 100% biochemical similarity to one another, respectively. The greater sequence divergence of the diatom POR enzymes compared to those in land plants likely reflects the ancient origins of the diatom POR enzyme duplication event, pre-dating the formation of extant stramenopile (and haptophyte) algal lineages ~800–1100 million years ago [6,8,41].

Both the smaller-than-expected protein detected by the POR2 antibody (44kD instead of 59kD) as well as the C-terminal extension found on the POR2 protein remain enigmatic. Though biochemical evidence is needed to confirm our hypothesis, we suggest that the unusual ~15kD C-terminal extension of the *P*. *tricornutum* POR2 protein may be cleaved, resulting in a protein of more conventional POR enzyme size. A *P*. *tricornutum*-like 15kD C-terminal extension is also present in the POR2 proteins of the diatoms *Fragilariopsis cylindrus*, *Pseudo-nitzschia multiseries*, *Thalassiosira pseudonana* as well as the pelagophyte stramenopile *Aureococcus anophagefferens* (accessions in [3]). These algae all share the same duplication of a horizontally-transferred prasinophyte *por* gene [3]. The C-terminal extensions of the diverse diatom POR2s show 50–68% biochemical similarity to one another and 39–50% similarity to that of *A*. *anophagefferens*. Extensive bioinformatic assessment as well as protein modeling was unsuccessful in assigning a function to this domain.

### Comparison of physiological responses at various light intensities

Repetitive sampling of *P*. *tricornutum* cultures maintained at either _200_L/D or _1200_L/D over several days, revealed moderately to tightly controlled oscillations in cell division response, cell size variation and chlorophyll accumulation. Phased cell division occurred at least once daily, usually taking place from the middle of the light interval to the middle of the dark interval. Cellular chlorophyll levels were ~70% higher in moderate (_200_L/D) than high light (_1200_L/D) cultures, reflecting the increased chlorophyll complement needed for effective light capture at reduced light fluences [22–24]. Upon transition of _200_L/D cultures to constant illumination (_200_L/L), the periodicity of cell division was disrupted and growth thereafter trended towards constant cell division [20,42], reduced cell size and a decreased rate of chlorophyll accumulation. In contrast, the first day of transition from _1200_L/D (high light) to _50_L/D (very low light) caused a rapid decline in cell division but maintenance of cellular chlorophyll levels. Over the next 6 days, the _50_L/D culture adjusted to the reduced light program by maintaining a reduced cellular division rate and diminished average cell size, while maintaining a steadily increasing cellular chlorophyll content. The decline of average cell size as *P*. *tricornutum* transitioned from high to low light fluences is noteworthy given that light absorption efficiency has been shown to be greater in smaller cells (reviewed in [43]).

### Regulatory differences among *por*1/POR1 and *por*2/POR2 suggest different roles for each protochlorophyllide oxidoreductase

Collectively, experiments presented here provide evidence that the *P*. *tricornutum por*1 and *por*2 paralogs are under different regulatory networks at both the transcriptional and post-translational levels. Cells acclimated to 12h light: 12h dark diurnal cycles (both _200_L/D or _1200_L/D) reveal a difference in timing between the daytime maxima and nighttime minima of *por*1 and *por*2 mRNA abundances. Furthermore, changes in photoperiod or light intensity markedly influenced *por*1/POR1 regulation whereas *por*2/POR2 regulation appeared robust. The transfer from a light:dark photoperiod to constant light altered *por*1 transcription and caused POR1 proteins to essentially disappear. In contrast, *por*2 mRNA and POR2 protein abundance continued to maintain a diurnal-like oscillation under continuous light despite lacking a dark interval cue.

Similar evidence for differential regulation of *P*. *tricornutum*’s *por* genes was documented in a microarray study performed under continuous (non-diurnal) lighting conditions [44]. Here a diatom culture was transferred from moderate light (100μE m^−2^ s^−1^) to 48 hours of darkness, followed by re-exposure to moderate light. Although *por*1 transcription was not down-regulated in prolonged darkness, reduced transcription of this gene occurred upon re-exposure to light. In contrast, *por*2 transcript abundance declined during dark treatment and only increased after several hours of re-exposure to light [44]. No companion protein study was done.

One may hypothesize that the POR1 enzyme plays a key role in photoacclimation, given the sensitivity of por1/POR1 regulation to changing light intensities in contrast to the apparent adherence of *por*2/POR2 regulation to the diurnal cycle. Indeed, in the present study, POR1 enzyme abundance increased to two and a half times the peak levels observed under _1200_L/D by the end of the first _50_L/D light interval, and thereafter remained elevated as a percentage of cellular protein, while POR2 protein abundance oscillated normally during the high to low light transition. The comparatively high abundance of *por*1 transcripts in dark-adapted cultures [44] and our finding that the regulation of *por*1/POR1 is responsive to real-time environmental changes is intriguing. In terms of cell survival, it would be interesting to explore whether POR1 plays a role in diatom photoacclimation when sediment-dwelling resting cells return to the photic zone [45]. Cumulatively, our results suggest that POR1 enables *P*. *tricornutum* to rapidly respond to changing environmental parameters. In contrast, the regulation of POR2 is intimately tied to the diurnal cycle and thus this enzyme may function as the ‘workhorse’ of chlorophyll production. Such complimentary roles of the diatom POR isozymes may enable diatoms to fine tune chlorophyll synthesis for both cell growth and expeditious response to shifts in environmental conditions.

In addition to light quantity, future studies should also consider light quality when exploring differential transcriptional responses of *por* genes. Valle et al. [46] showed similar levels of *por*1 transcription in dark-adapted *P*. *tricornutum* cells exposed for 0.5h to photosynthetically equivalent quantities of red, green, blue and white light. In conjunction with our results, one may speculate that light intensity or duration rather than quality may influence the regulation of the *por*1 gene. In contrast, *por*2 transcription was down-regulated under blue light and up-regulated under red and green light. A potential link between *por*2 transcription and blue light reception is especially intriguing given the universal roles played by blue-light receptors such as cryptochromes in the entrainment and maintenance of circadian rhythms [47,48] and the strict adherence to diurnal light cues we observe for *P*. *tricornutum por*2 transcription as well as cellular POR2 abundance. Further work is warranted to determine whether *por*2 is regulated by a cryptochrome or other blue-light receptor such as an aureochrome (a class of photoreceptor specific to the diatom crown-taxon Stramenopila [49]). Notably, silencing of specific *P*. *tricornutum* aureochromes has been shown to effect a high-light acclimation-like phenotype that has reduced chlorophyll *a* levels [50].

Future studies must also account for potential discordance between transcriptional and post-translational regulation of chlorophyll synthesis in diatoms. Although *P*. *tricornutum por*2 mRNA transcription showed a similar pattern to POR2 cellular protein abundance in our studies, *por*1 transcript abundance did not match observed levels of POR1 protein (Figs 6 and 8). Our observations caution that studying chlorophyll biosynthesis solely at the level of gene transcription might be misleading, a lesson also gleaned from studies of terrestrial plants. For example, despite nearly constitutive *por* mRNA transcription, abundance of the sole POR protein of *Pisum sativum* (pea) declines upon exposure of etiolated seedlings to light [51]. Light-induced proteases were found to be responsible for a similar loss of PORA from etiolated seedlings of *H*. *vulgare* [52]. In addition to targeting the POR enzyme itself, post-translational regulation of chlorophyll synthesis in plants involves a highly reticulated metabolic network that modulates POR substrate availability (reviewed in [53,54]). Given physiological and developmental differences among land plants and algae, it will be interesting to further explore post-translational regulation of chlorophyll production in diatoms.

Exploring beyond enzyme regulation, gene silencing and gene editing techniques recently developed for diatoms [25–27] may enable studies probing enzyme function. For example, it would be of interest to know if *P*. *tricornutum* POR1 and POR2 enzymes could catalytically replace one another (i.e., could a cell survive with only one POR isozyme?). The POR enzyme is a member of the short-chain dehydrogenase-reductase (SDR) family of proteins, the majority of which are found as dimers and tetramers. This observation brokers the possibility for both homo- and hetero-complexes when isoenzymes are present [55,56]. Indeed, oligomerization has been reported for some PORs [57,58]. The role of the conserved C-terminus of POR2 remains an interesting target for further exploration.

### Comparison of *por* gene duplicate regulation among diverse taxa

As noted above, *por* gene expansions have been well studied in land plants [1,4] and recently documented in a broad representation of algal taxa [3]. Whether the regulatory and functional roles of *por* gene sets have converged in response to similar environmental stimuli or diverged in response to different physiologies, ecologies, and stochastic effects during evolution remains undeciphered.

Interestingly, *P*. *tricornutum*’s *por*1 and *por*2 regulatory schemes appear to be similar in some ways to those documented for the land plant *A*. *thaliana por*C *and por*B genes, respectively [17]. Both *P*. *tricornutum por*1 and *por*2 and *A*. *thaliana por*B and *por*C are under diurnal regulation. *P*. *tricornutum por*2 and *A*. *thaliana por*B are potentially under circadian control and their expression appears unaffected by light intensity. The regulatory responses of *P*. *tricornutum por*1 and *A*. *thaliana por*C, on the other hand, are greatly affected by changes in light intensity–though most likely differently. Because patterns of chlorophyll accumulation in response to light intensity contrast in algae and land plants (algae decrease and land plants augment chlorophyll abundance under increased light levels [21–24]), we anticipated that the *P*. *tricornutum por*1 and *A*. *thaliana por*C enzymatic responses would be inverted. Indeed, *A*. *thaliana por*C is up-regulated with increasing light intensities [17], whereas *P*. *tricornutum* POR1 levels rise upon transfer to low light. Note that many *por* regulatory schema divergent from that of *A*. *thaliana* (and *P*. *tricornutum*) have been documented in other plant species (e.g., [4,16,18,59,60]).

In contrast to terrestrial plants, little data concerning *por* gene set regulation and enzyme function is available for photosynthetic protists. What minimal data is available suggests that regulatory patterns for the *por* genes found among taxa differ. For example, transcriptomic analyses indicate that the diurnal regulatory programs observed for *Chrysochromulina tobin* (haptophyte) *por*1 and *por*2 genes differ significantly from those of *P*. *tricornutum* [61]. Additional transcriptomic data suggest a seminal role for *por*2 in the life history transition of stramenopile *Heterosigma akashiwo* (raphidophyte), as the alga transitions from a dark-dwelling resting phase to an active, photosynthesizing state [62]. It is interesting to note that a diversity of protochlorophyllide reduction strategies exists within stramenopiles. Some taxa maintain both *por* gene duplicates (and occasionally an additional copy of one duplicate, as in the diatom *Fragilariopsis cylindrus*) but lack LIPOR (the light-independent protochlorophyllide oxidoreductase), whereas other taxa possess solely one *por* gene plus LIPOR [e.g., *Chattonella subsalsa* (Raphidophyceae), *Ectocarpus siliculosus* (Phaeophyceae), *Pinguiococcus pyrenoidosus* (Pinguiophyceae)] [3]. It will be of interest to compare the conditions promoting POR versus LIPOR expression in these diverse algal taxa.

## Conclusions

- This study represents the first paired analysis of transcriptional and post-translational differences between *por* gene duplicates in an alga.
- The regulatory networks governing each *P*. *tricornutum por* gene appear to be distinct given their different responses to light cues. Data suggest a role in photoacclimation for *por*1 and in daily chlorophyll homeostasis for *por*2.
- For the vast majority of algal crown taxa, the regulatory and functional schemes of their diverse *por* gene sets await characterization. This report provides a template for probing regulatory schemes of *por* gene expansions documented in a diverse array of algal taxa.
- Comparisons among algae as well as between algae and terrestrial plants will lead to interesting insights concerning the fate of homologous gene duplicates across taxa varying in their ecologies, physiologies, and evolutionary histories.

## Materials and Methods

### Culture growth

*Phaeodactylum tricornutum* Bohlin CCMP632 stock cultures were grown in 1L f/2 medium [63] contained in 2.8L fernbach flasks that were stoppered with cotton and gauze plugs, capped with sterilizer bags, and shaken at 60rpm. Stock cultures were maintained at 20°C and 100μE m^-2^ s^-1^ on a 12 hour light: 12 hour dark photocycle and transferred every 4–5 days to maintain cells in the exponential growth phase. For experimental studies, *P*. *tricornutum* exponential growth phase cultures were used to inoculate an autoclaved 15L MicroFerm Fermentor (New Brunswick Scientific: Edison, NJ) that contained 12L sterile f/2 medium. Starting cell density was 2.5x10^5^ cells/mL for the _200_L/D-_200_L/D experiment and 5x10^3^ cells/mL for the _1200_L/D-_50_L/D study. The 12L culture was maintained at ~17°C using a VWR 1160 re-circulating water chiller (Radnor, PA). Air provided to the culture (1000 cc/min) was first filtered through a Millex 50mm hydrophobic PFTE 0.2μM in-line filter (EMD Millepore: Billerica, MA) to remove potential bacterial contaminates, then bubbled through sterilized water. The culture was mixed using a custom right-handed (upward mixing) impeller with 4 blades pitched at 45° and overall diameter of 10.6cm (culture vessel of diameter 22.9cm) turning at 50rpm. The photoreactor was illuminated on all lateral surfaces with Xlamp XP-E cool white LEDs (Cree: Durham, NC) operated by a custom, programmable controller. External light was excluded by fitting the culture unit and ~30cm of the bases of its air, media and collection tubes with a cover made of black-out duvetyne fabric (Filmtools, Burbank, CA). Upon reaching experimental density, the cultures were maintained in exponential growth by daily dilution at the beginning of the light period (L0).

### Sampling

In addition to a ~3.0mL sample used for cell counts, 12 tubes containing 45mL of culture were collected at each time point for RNA and protein studies. These samples were kept on ice until centrifugation at 6000x*g* for 20min at 4°C. The samples were then decanted, the pellet flash frozen in liquid nitrogen and stored at -80°C. Samples harvested during a ‘dark’ portion of the light/dark cycle were collected and decanted under dim green light provided by Cree Xlamp XP-E green LEDs in a room protected from external light via a duvetyne drape. The light- and dark-harvested samples were collected into clear 50mL (VWR, Radnor, PA) or black 50mL LiteSafe conical centrifuge tubes (Argos Technologies, Elgin, IL), respectively.

### Determination of culture density, chlorophyll abundance and cell size

Culture density was monitored in a BD Accuri C6 flow cytometer (San Jose, CA) using cellular fluorescence to differentiate *P*. *tricornutum* cells from debris. Approximately 80μL of culture was measured in duplicate for each time point and resultant values averaged. An additional sample was analyzed if the initial results were more than ~5% different. Cellular fluorescence (FL-3 channel; excitation 488nm; emission 670nm long pass) was used to estimate *P*. *tricornutum* cellular chlorophyll *a* content, as HPLC [17] and spectrophotometric (S3 Fig) measurements of chlorophyll *a* levels show excellent concordance between cellular fluorescence and chlorophyll *a* content. Flow cytometric measurements of cell size were performed with the Life Technologies flow cytometry size calibration kit (Grand Island, NY) using the FSC-H measurement of particle size. Given the elongate (fusiform) shape of *P*. *tricornutum*, size estimates likely refer to the width of cells as they travel single-file past the electronic detectors, with increased width indicating dividing cells.

### Determination of gene expression

#### cDNA preparation

RNA was extracted from cell pellets thawed on ice using TRIzol reagent (Life Technologies) according to manufacturer’s directions with 1mL TRIzol per 2x10^8^ cells. Recovered RNA was quantitated with a NanoDrop UV-vis spectrophotometer (Thermo Fisher Scientific, Waltham, MA), diluted to less than 20μg per 100μL, and treated with DNase I (12U per 10μg RNA) at 37°C for 30min in the presence of 1X DNase I buffer (Life Technologies). The RNA was then further purified with the RNeasy MinElute Cleanup Kit (Qiagen, Valencia, CA) according to manufacturer’s directions. To further ensure RNA quality, RNA was tested for DNA contamination by performing qPCR with RNA extracts using 2-4X the amount of RNA as the amount of cDNA used in normal reactions. A ΔC_t_ of 5 between the amplification of DNA-contaminated RNAs and the highest experimental C_t_ for primer *TBP* (as determined in preliminary experiments) represents 3.1% contamination (or 0.8–1.6% when accounting for the greater amount of starting nucleic acid), and this level of contamination was considered acceptable for RT-qPCR studies. RNAs demonstrating higher levels of DNA contamination were re-DNAsed, purified, and tested again. RNA was reverse-transcribed to cDNA using the Bio-Rad iScript cDNA Synthesis Kit (Hercules, CA) in 20μL reactions each containing 1μg RNA.

#### RT-qPCR

Primer sets of four reference genes identified by Siaut et al. [39] for use in diurnal cycle studies in *P*. *tricornutum* were tested: *cdkA* (cyclin-dependent kinase A), *H4* (histone), *RPS* (30S ribosomal protein subunit), and *TBP* (TATA box binding protein). Only *cdkA* and *TBP* (not *RPS* or *H4*) showed low standard deviations (± CP and ± x-fold) and high coefficients of correlation (r) when analyzed with BestKeeper reference gene evaluation software ([40]; S4 Fig). The *por*1 and *por*2 primers were designed in Primer3 [64] as per Siaut et al. [39]. Amplicon sizes were verified by gel electrophoresis and amplification efficiencies assessed by qPCR (S1 Table). Reactions were performed on a Chromo4 Real-Time PCR system with iQ SYBR Green Supermix (Bio-Rad) in white 96-well plates with optically clear seals (Bio-Rad) using the following program: initial denaturation was at 96°C for 5min, followed by 40 cycles of 30sec denaturation at 96°C; 30sec annealing at 60°C and 30sec extension at 72°C, then a melting curve from 60–95°C with 10sec holds at each 0.5°C interval. Triplicate 20μL reactions were performed with 5ng of cDNA (2μL of 1/20 dilutions) and 0.1μM (*TBP*, *H4*), 0.2μM (*cdkA*, *por*2, *RPS*), or 0.3μM (*por*1) primer. A 4-fold dilution series from 100ng to 0.024ng cDNA was amplified simultaneously for the determination of reaction efficiency as per [65]. Negative controls were included to ensure that PCR reagents were not contaminated. Data were analyzed with the Bio-Rad Gene Expression Analysis macro which incorporates primer amplification efficiency and reference gene expression according to the method of Vandesompele and colleagues [66].

### Antibody preparation

#### 3’ RACE of *por*1 and *por*2 cDNAs

Aliquots of *P*. *tricornutum* 1L cultures growing exponentially at 100μmol photons m^-2^ s^-1^ and 20°C on a 12h light: 12h dark photoperiod were harvested at ~L7 by centrifugation at 5000x*g* for 15min at 4°C. The recovered pellets were flash frozen in liquid nitrogen. RNA was extracted, treated with DNAse, and purified as described above. cDNA synthesis was carried out using the SuperScript III First-Strand Synthesis System (Life Technologies) using 1.2μg RNA per 20μL reaction. The following reaction was used to amplify *por*1 and *por*2 cDNAs: 1X Phusion HF buffer and 0.02U/μL Phusion High-Fidelity DNA polymerase (New England Biolabs, Ipswich, MA), 200μM dNTPs (Lamda Biotech, St. Louis, MO), 0.5μM each forward and reverse primers, and 1μL cDNA (from the above reverse transcription) in 25μL reactions. A universal primer that anneals to the polyA tail of the mRNA ([67]) or nested 5’ primers based on the 5’ regions of *por*1 and *por*2 ESTs from Maheswari et al. ([68]; S1 Table) were used in the cDNA synthesis reactions. Cycling reactions were performed in an Eppendorf Mastercycler gradient thermocycler (Hauppage, NY) as follows: initial denaturation was at 98°C for 30sec, followed by 35 cycles of 10sec denaturation at 98°C, 20sec annealing at 56°C, and a 1min extension at 72°C, then 5min final elongation at 72°C. Prior to sequencing, reactions were further treated by the addition of Exonuclease I (0.2U)/shrimp alkaline phosphatase (0.08U) and incubated at 37°C for 45min, then 85°C for 15min (Affymetrix, Santa Clara, CA). Sequencing was performed on an ABI 3130xl Genetic Analyzer using the ABI BigDye Terminator v3.1 Cycle Sequencing kit with 1/8^th^ the manufacturer’s recommended reaction size (Applied BioSystems, Foster City, CA).

#### *P*. *tricornutum* POR1 and POR2 heterologous expression constructs

*Por*1 and *por*2 cDNAs were re-amplified using Phusion High-Fidelity Polymerase and primers designed to enable Gibson Assembly ([69]; S1 Table) according to PCR protocols described above. The pET-15-HE vector (obtained from Dr. Stoddard, Seattle Children’s Hospital, Seattle, WA) was digested by incubation at 37°C for 2h in a 40μL reaction containing 3μg vector, 7.5U each of NcoI, NotI enzymes and 1X NE Buffer 3 (New England Biolabs). The linearized vector and PCR inserts were each purified with the QIAquick PCR Purification kit (Qiagen). Digested vector (50ng) and either *por*1 or *por*2 PCR inserts (50ng) at a ratio of ~1M vector:3M insert were incubated with 20μL Gibson Assembly MasterMix (New England Biolabs) at 50°C for 1h. A reaction containing 50μL competent *Escherichia coli* DH5α (Life Technologies) and 5μL of the Gibson Assembly product were incubated on ice for 30min, heat shocked at 42°C for 45sec, then cooled on ice for 2min. Luria Broth medium (500μL) was added to the reaction mixtures prior to incubation at 37°C for 1h with shaking at 200rpm. Transformed cells were spread on Luria Broth agar plates (1.5% Bacto agar; Becton Dickinson & Co., Franklin Lakes, NJ) containing 100μg/mL carbenicillin (Sigma-Aldrich) and grown overnight at 37°C. Individual colonies were picked and suspended into 5mL of Luria Broth medium containing 100μg/mL carbenicillin and grown at 37°C overnight with shaking at 200rpm. The QIAprep Spin MiniPrep Kit was used to purify plasmid DNA (Qiagen). GENEWIZ DNA sequencing services (Seattle, WA) were used to sequence plasmid DNAs using an upstream T7 and an internal primer (S1 Table) to verify sequence identity and orientation. The above protocol was then used to transform and plate *E*. *coli* c2566 (New England Biolabs) with each plasmid.

#### POR1 and POR2 heterologous expression

Protein expression in *E*. *coli* c2566 was found to be leaky and growth overnight at 30°C without IPTG induction produced copious quantities of POR1 and POR2 proteins (Fig 3). Overnight cultures were centrifuged at 2000xg for 20min at 4°C, and re-suspended in 10% the original culture volume of lysis buffer [PBS (50mM sodium phosphate, 300mM sodium chloride, pH 7) containing 0.1% Triton-X and 5mM β**-**mercaptoethanol and cOmplete ULTRA EDTA-free protease inhibitors (1 tablet per 10mL; Roche, Nutley, NJ)]. Additionally, 6M guanidine hydrochloride was used to ensure solubility of POR2. Upon addition of 1mg/mL lysozyme (Sigma-Aldrich, St. Louis, MO) and incubation on ice for 30min, the lysates were sonicated at 4°C for 3min in 10sec intervals with 30sec pauses. Supernatants containing soluble POR1 or POR2 enzymes were recovered after centrifugation at 12000xg for 25min at 4°C.

#### Affinity purification

POR1 and POR2 enzymes were subject to affinity purification using the 6X histidine tag encoded in the pET-15-HE vector (Fig 3). The Clontech TALON Metal Affinity Resin system was used according to manufacturer’s protocols using 2mL resin per 25mL lysis buffer containing bacterial cell pellet from 250mL culture (Mountain View, CA). Fractions were evaluated by SDS-PAGE, and those containing purified protein were concentrated in Millipore Amicon Ultra centrifugal filter units per manufacturer’s directions.

#### Antibody preparation

Antibodies were prepared by Yenzym (San Francisco, CA). Anti-POR1 antibody was generated in a chicken and anti-POR2 antibody raised in a pre-screened rabbit. The POR1 antibody was affinity purified using ThermoScientific Pierce NHS-Activated Agarose slurry according to manufacturer’s directions, with 6mg POR1 protein coupled to 2mL slurry. The specificity of the POR2 antibody was verified by comparing the cross-reactivity of un-blocked antibody with that of antibody blocked by incubation at 4°C overnight with 10X (w/w) POR2 affinity purified protein (Fig 3H and 3I).

### POR1 and POR2 protein quantitation

#### Protein extraction and quantitation

Triplicate protein extractions were performed for each time point according to the methanol/chloroform/water method of Wessel and Flugge [70]. Unless indicated, all procedures were performed at room temperature. Briefly, each extraction used pellets from two 45mL samples. One mL methanol was added to ~250μL of loose pellet to attain an approximately 80% methanol concentration.

Upon vortexing on high for 60sec, the contents of two tubes were transferred to one 15mL conical centrifuge tube. Chloroform (500μL) was added to the combined solutions, which were vortexed for 60sec at room temperature. Water was added (1.5mL) and the sample vortexed another 30sec. Phases were separated by centrifugation for 5min at room temperature. After removal of the upper chloroform phase, the protein containing interface and bottom phase were transferred to a 2mL centrifuge tube. A 1.5mL methanol rinse of the extraction tube was then added to the 2mL tube. The solution was vortexed for 10sec. Precipitated protein was recovered by centrifugation at 14600xg for 10min at 4°C. After removal of the supernatant, the pellet was dried by SpeedVac for 5min. Samples were re-suspended in 150μL DIGE buffer (7M urea, 2M thiourea, 30mM Tris-base, 4% CHAPS, pH 8.5) that contained Roche cOmplete ULTRA EDTA-free protease inhibitors (1 tablet per 10mL). After the samples were gently vortexed for 1h, non-solubilized material was removed by centrifugation at 14600xg for 12min at 4°C. Protein in the supernatant was quantitated using the Life Technologies EZQ protein extraction kit. Preliminary experiments showed that under these re-solubilization conditions, equal protein extraction efficiency was attained for samples regardless of the upper and lower cell concentration used in these studies.

#### Western blotting

For each time point, 4μg protein was denatured for 10min at 70°C with 1X NuPage LDS sample buffer and 1X NuPage reducing agent (Life Technologies). Proteins were separated on pre-cast NuPage Novex 4–12% Bis-Tris protein gels (Life Technologies) run in MOPS buffer (50mM MOPS, 50mM Tris-base, 0.1% SDS, 1mM EDTA). SeeBlue Plus2 pre-stained protein standard (Life Technologies) served as molecular markers for protein size determination. Proteins were transferred to nitrocellulose (POR1) or PVDF (POR2) membranes using the iBlot gel transfer device and transfer stacks (Life Technologies). After blocking for 1h at 20°C with 5% nonfat milk in TBST buffer (20mM Tris, 150mM NaCl, pH 7.6, 0.1% Tween-20), blots were incubated overnight at 4°C with anti-POR1 (1:600 in TBST with 2% milk) or anti-POR2 (1:7500 in TBST with 2% milk) antibodies. After three 10min washes in TBST, blots were incubated for 1h at 20°C with HRP-conjugated anti-chicken antibodies (1:20000 in TBST with 2% milk; Abcam, Cambridge, MA) or HRP-conjugated anti-rabbit antibodies (1:6000 in TBST; GE Amersham, Pittsburgh, PA). Following two 10min washes in TBST and one 10min wash in TBS (TBST without Tween-20), the blots were incubated with SuperSignal West-Pico chemiluminescent substrate (Life Technologies) and visualized for 2min (POR1) or 30sec (POR2) on X-ray film (Phenix Research, Candler, NC). Band intensities were quantitated in ImageJ [71] for each of three to four replicate blots. As each blot contained all time points, data were normalized within each blot such that the total density across time points summed to 100. Membranes were stained with Coomassie Brilliant Blue to check for equal loading.

#### Statistical analyses

Diurnal cycling of POR2 abundance per μg protein and per cell was analyzed with paired Welch’s unequal variance t-tests. Light interval means were paired with dark interval means from _50_L/D, _200_L/D, and _1200_L/D, and the analysis was repeated when also including the _200_sD_3_/_200_L_4_ pair. A two-way ANOVA was performed to compare POR1 abundance per μg protein in the light and dark intervals of _1200_L/D and _50_L/D. A one-way ANOVA followed by a Tukey’s HSD test was used to compare POR1 abundance per μg protein among all time points in _1200_L/D and _50_L/D.

## Supporting Information

S1 Fig 15L photobioreactor with LED lighting. (PDF)

S2 Fig Light-intensity dependent growth of *P*. *tricornutum*. (a) Comparison of growth for *P*. *tricornutum* batch culture at 200μE m^-2^ s^-1^ on a 12h light: 12h dark photoperiod (_200_L/D); batch culture under constant illumination at 200μE m^-2^ s^-1^ (_200_L/L); and under the semi-continuous experimental culture conditions of Figs 5 and 6 (_200_L/D-_200_L/L). (b) Comparison of growth for *P*. *tricornutum* batch cultures at 50μE m^-2^ s^-1^ on a 12h light: 12h dark regime (_50_L/D); batch culture at 1200μE m^-2^ s^-1^ on a 12h light: 12h dark regime (_1200_L/D); and under the semi-continuous experimental culture conditions of Figs 7 and 8 (_1200_L/D-_50_L/D).
(PDF)

S3 Fig Relationship between flow cytometric measurements of cellular fluorescence and spectrophotometric measurements of chlorophyll *a* in *P*. *tricornutum*. Trendline R^2^ value of 0.89 indicates a linear relationship between cellular fluorescence and chlorophyll *a* content determined by spectrophotometric assessment. Replicate values are plotted separately for samples from cultures maintained at 50μE m^-2^ s^-1^, 200μE m^-2^ s^-1^, or 1200μE m^-2^ s^-1^ that were subject to either 12h light: 12h dark (L/D) or constant illumination (L/L). Samples were obtained at D11 (L/D only), L2, and L6 time points. Arrows indicate two data points excluded from linear regression.
(PDF)

S4 Fig Reference gene expression. **(**a) Reference gene expression over the course of the _200_L/D:_200_L/L experiment. (b) Experimental gene expression over the course of the _200_L/D:_200_L/L experiment. (c) Reference gene expression over the course of the _1200_L/D:_50_L/D experiment. (d) Experimental gene expression over the course of the _1200_L/D:_50_L/D experiment.
(PDF)

S1 Table RT-qPCR, 3’ RACE and cloning primers unique to this study. (PDF)
