# Supplementary material for: Differential Regulation of Duplicate Light-Dependent Protochlorophyllide Oxidoreductases in the Diatom Phaeodactylum tricornutum
Source: PLoS One. 2016 Jul 1;11(7):e0158614. doi: 10.1371/journal.pone.0158614 (PMC4930169; doi:10.1371/journal.pone.0158614)
Supplement: S1 Fig — (PDF) [file pone.0158614.s001.pdf]

S1 Fig: 15L photobioreactor with LED lighting.

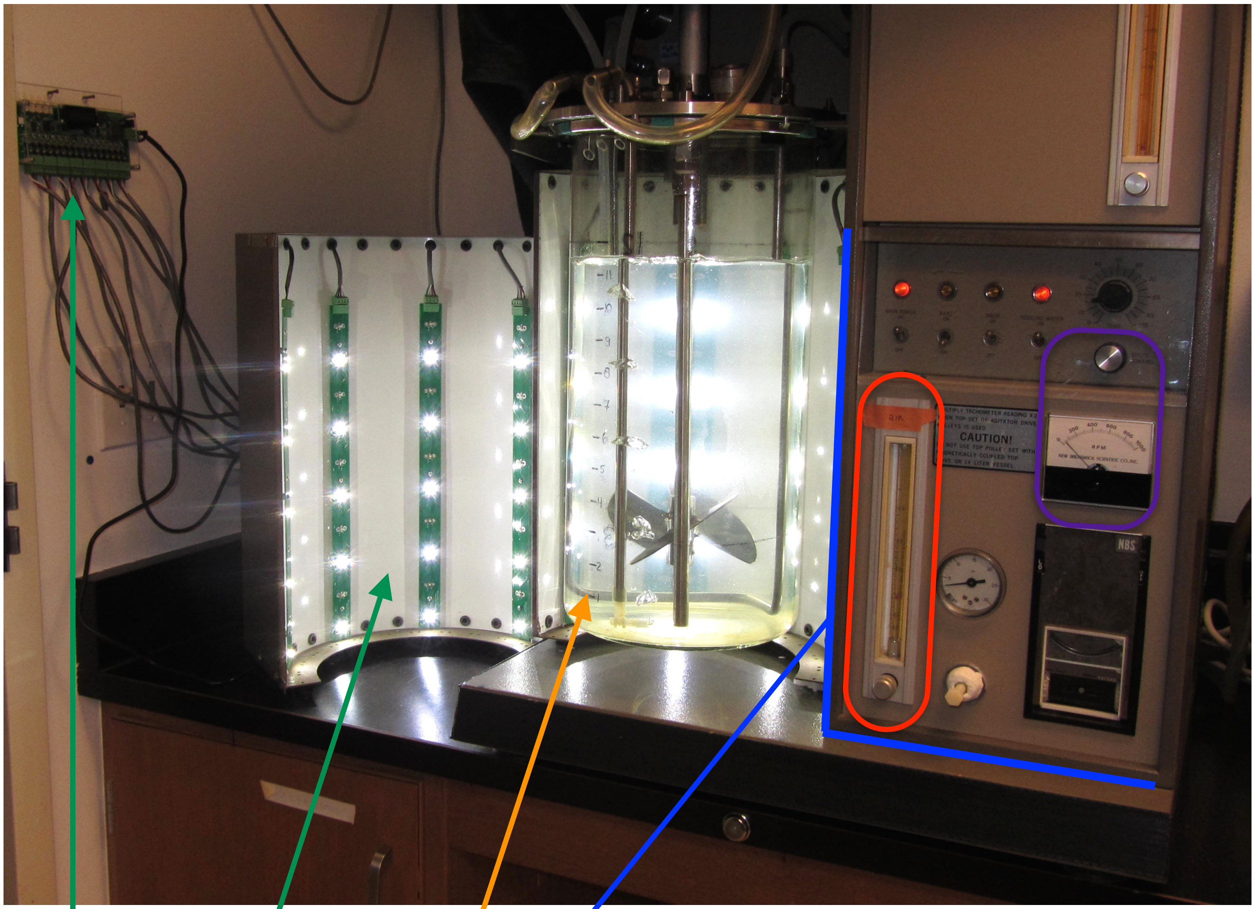

MicroFerm Fermentor (New Brunswick) control panel, including:

Agitation speed (rpm) with attached custom, upward mixing impeller (right-handed, 4 blades at 45°).

Aeration rate (cc/min). Before reaching the 'fermentor', air was filtered through a Millex 50mm hydrophobic PFTE 0.2µM in-line filter (not pictured) and hydrated by bubbling through sterile water (not pictured).

Cooling coil running through 15L tank, filled to 12L. Cold water was fed from VWR 1160 re-circulating water chiller (not pictured), with pumping provided by both the 'fermentor' and water chiller units.

Custom LED control board regulates eight LED lighting strips encircling tank. Each strip is subdivided into two banks of four LEDs with bank intensity programmable from 0-100% in 0.5% increments.
