# Supplementary material for: Differential Regulation of Duplicate Light-Dependent Protochlorophyllide Oxidoreductases in the Diatom Phaeodactylum tricornutum
Source: PLoS One. 2016 Jul 1;11(7):e0158614. doi: 10.1371/journal.pone.0158614 (PMC4930169; doi:10.1371/journal.pone.0158614)
Supplement: S2 Fig — (a) Comparison of growth for P. tricornutum batch culture at 200μE m-2 s-1 on a 12h light: 12h dark photoperiod (200L/D); batch culture under constant illumination at 200μE m-2 s-1 (200L/L); and under the semi-continuous experimental culture conditions of Figs 5 and 6 (200L/D-200L/L). (b) Comparison of growth for P. tricornutum batch cultures at 50μE m-2 s-1 on a 12h light: 12h dark regime (50L/D); batch culture at 1200μE m-2 s-1 on a 12h light: 12h dark regime (1200L/D); and under the semi-continuous experimental culture conditions of Figs 7 and 8 (1200L/D-50L/D). (PDF) [file pone.0158614.s002.pdf]

S2 Fig: Light intensity-dependent growth of *P. tricornutum*

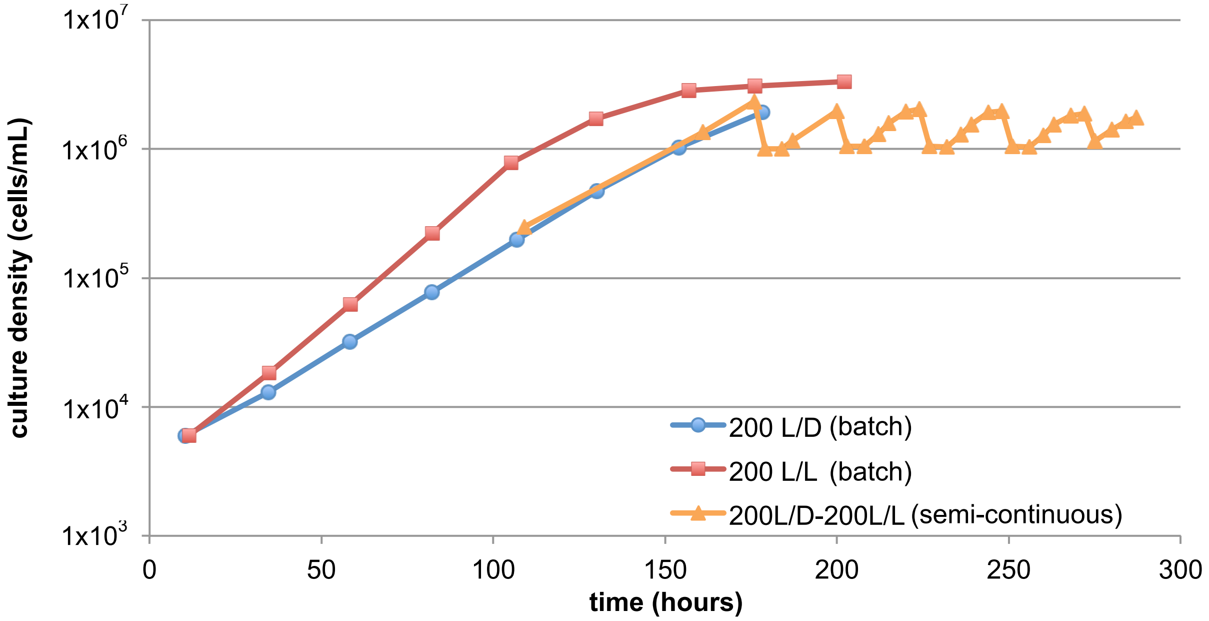

(a) Comparison of growth for *P. tricornutum* batch culture at 200μE m^{-2} s^{-1} on a 12h light: 12h dark photoperiod ($_{200}$L/D); batch culture under constant illumination at 200μE m^{-2} s^{-1} ($_{200}$L/L); and under the semi-continuous experimental culture conditions of Figs 5 and 6 ($_{200}$L/D-$_{200}$L/L).

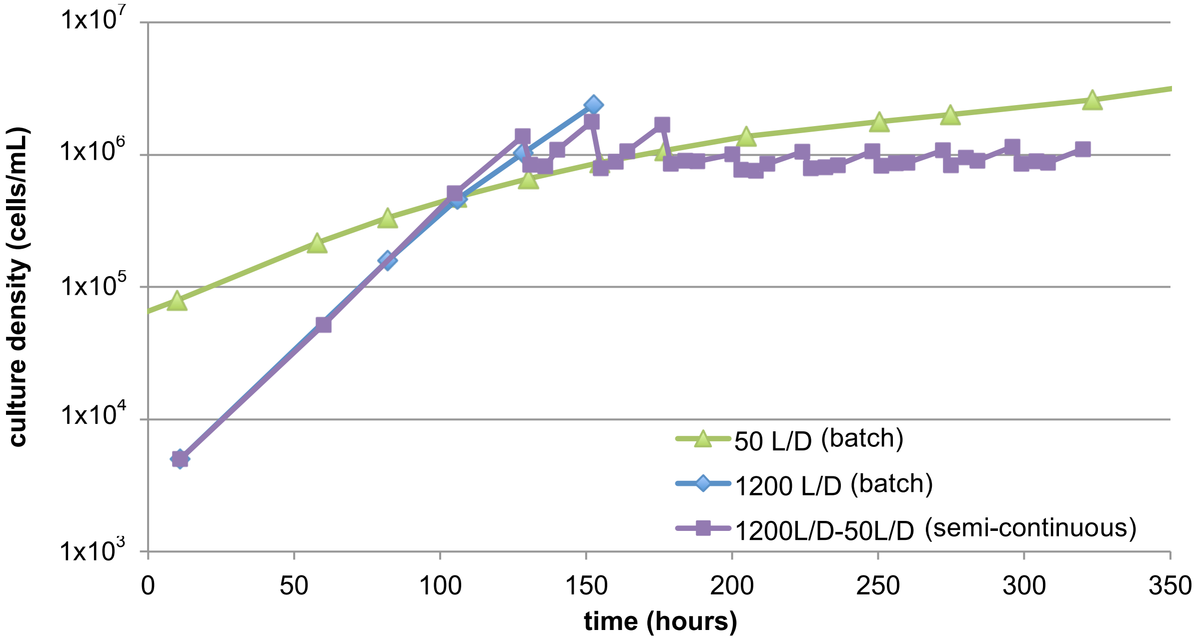

(b) Comparison of growth for *P. tricornutum* batch cultures at 50μE m^{-2} s^{-1} on a 12h light: 12h dark regime ($_{50}$L/D); batch culture at 1200μE m^{-2} s^{-1} on a 12h light: 12h dark regime ($_{1200}$L/D); and under the semi-continuous experimental culture conditions of Figs 7 and 8 ($_{1200}$L/D-$_{50}$L/D).
