# Supplementary material for: Differential Regulation of Duplicate Light-Dependent Protochlorophyllide Oxidoreductases in the Diatom Phaeodactylum tricornutum
Source: PLoS One. 2016 Jul 1;11(7):e0158614. doi: 10.1371/journal.pone.0158614 (PMC4930169; doi:10.1371/journal.pone.0158614)
Supplement: S3 Fig — Trendline R2 value of 0.89 indicates a linear relationship between cellular fluorescence and chlorophyll a content determined by spectrophotometric assessment. Replicate values are plotted separately for samples from cultures maintained at 50μE m-2 s-1, 200μE m-2 s-1, or 1200μE m-2 s-1 that were subject to either 12h light: 12h dark (L/D) or constant illumination (L/L). Samples were obtained at D11 (L/D only), L2, and L6 time points. Arrows indicate two data points excluded from linear regression. (PDF) [file pone.0158614.s003.pdf]

S3 Fig: Relationship between flow cytometric measurements of cellular fluorescence and spectrophotometric measurements of chlorophyll *a* in *P. tricornutum*.

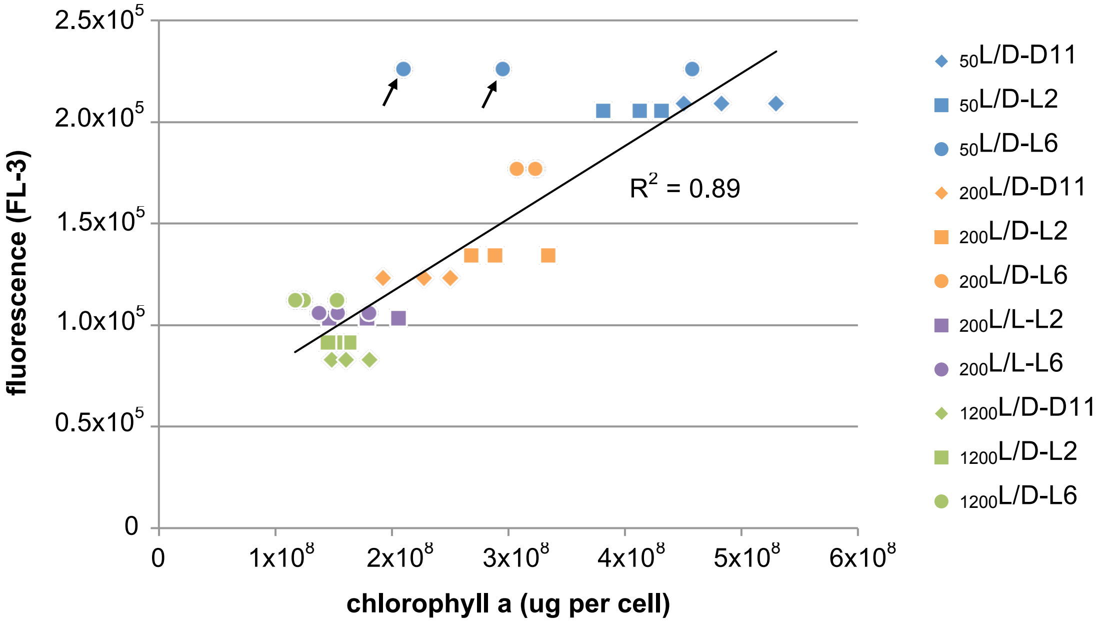

Trendline R^2 value of 0.89 indicates a linear relationship between cellular fluorescence and chlorophyll *a* content determined by spectrophotometric assessment. Replicate values are plotted separately for samples from cultures maintained at 50μE m^{-2} s^{-1}, 200μE m^{-2} s^{-1}, or 1200μE m^{-2} s^{-1} that were subject to either 12h light:12h dark (L/D) or constant illumination (L/L). Samples were obtained at D11 (L/D only), L2, and L6 time points. Arrows indicate two data points excluded from linear regression.

Methods:

Cell density and cellular fluorescence was measured on a BD Accuri C6 flow cytometer. Spectrophotometric measurements utilized flash-frozen pellets from 10mL of culture, extracted into 1.5mL 90% acetone via vortexing, 20min sonication in an ice bath, and 12h incubation at 4°C in the dark prior to centrifugation at 6000 x *g* to remove sediments. The equation of Jefferey and Humphrey [1] was utilized to determine the chlorophyll *a* content of cells.

Reference:

1. Jeffrey SW, Humphrey GF. New spectrophotometric equations for determining chlorophyll a, b, c1 and c2 in higher plants, algae and natural phytoplankton. Biochem Physiol Pflanz. 1975;167: 191–194.
