# Supplementary material for: Differential Regulation of Duplicate Light-Dependent Protochlorophyllide Oxidoreductases in the Diatom Phaeodactylum tricornutum
Source: PLoS One. 2016 Jul 1;11(7):e0158614. doi: 10.1371/journal.pone.0158614 (PMC4930169; doi:10.1371/journal.pone.0158614)
Supplement: S4 Fig — (a) Reference gene expression over the course of the 200L/D:200L/L experiment. (b) Experimental gene expression over the course of the 200L/D:200L/L experiment. (c) Reference gene expression over the course of the 1200L/D:50L/D experiment. (d) Experimental gene expression over the course of the 1200L/D:50L/D experiment. (PDF) [file pone.0158614.s004.pdf]

S4 Fig: Reference gene expression

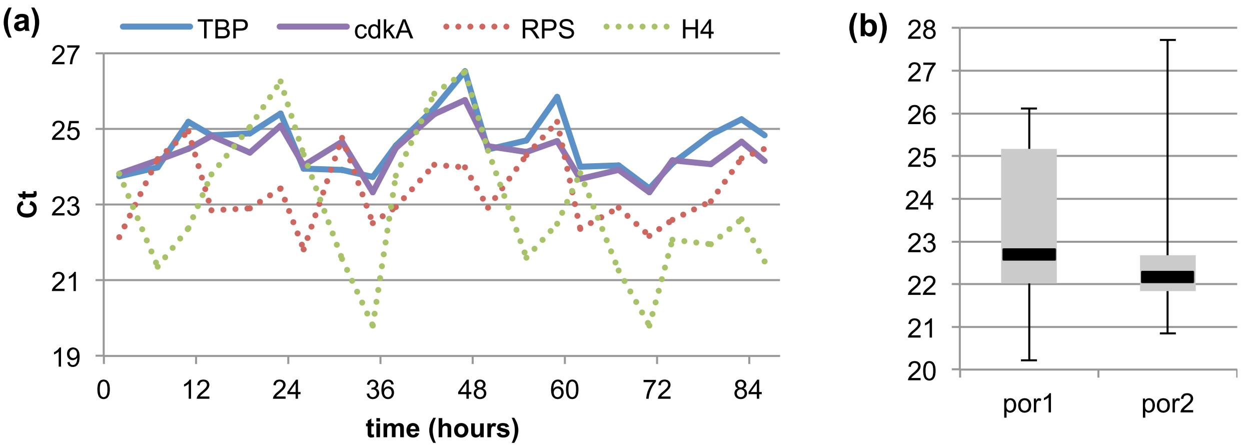

(a) Reference gene expression over the course of the $_{200}$**L/D:**$_{200}$**L/L** experiment.

	ΔC_t	BestKeeper output std dev (±CP)	std dev (±x-fold)	coeff corr (r)	*TBP* & *cdkA* only coeff corr (r)
TBP	3.1	0.63	1.57	0.895*	0.970*
cdkA	2.4	0.45	1.38	0.945*	0.950*
RPS	3.4	0.87	1.86	0.495	n/a
H4	6.7	1.61	3.15	0.828*	n/a

Data normalized to *TBP* and *cdkA* only. *RPS* and *H4* were not used. (*p<0.001)

(b) Experimental gene expression over the course of the $_{200}$**L/D:**$_{200}$**L/L** experiment.
ΔC_t *por*1=5.9; ΔC_t *por*2=6.9.

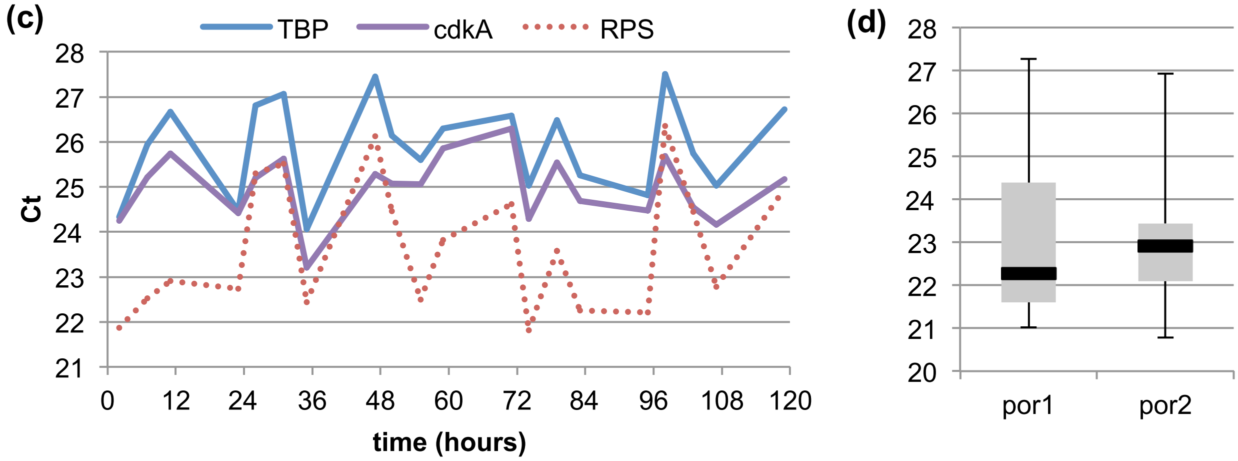

(c) Reference gene expression over the course of the $_{1200}$**L/D:**$_{50}$**L/D** experiment.

	ΔC_t	BestKeeper output std dev (±CP)	std dev (±x-fold)	coeff corr (r)	*TBP* & *cdkA* only coeff corr (r)
TBP	3.4	0.88	1.89	0.972*	0.971*
cdkA	3.1	0.59	1.53	0.819*	0.946*
RPS	4.5	1.27	2.51	0.973*	n/a

Data normalized to *TBP* and *cdkA* only. *RPS* was not used. (*p<0.001)

(d) Experimental gene expression over the course of the $_{1200}$**L/D:**$_{50}$**L/D** experiment.
ΔC_t *por*1=6.3; ΔC_t *por*2=6.1.
