# Supplementary material for: Differential Regulation of Duplicate Light-Dependent Protochlorophyllide Oxidoreductases in the Diatom Phaeodactylum tricornutum
Source: PLoS One. 2016 Jul 1;11(7):e0158614. doi: 10.1371/journal.pone.0158614 (PMC4930169; doi:10.1371/journal.pone.0158614)
Supplement: S1 Table — (PDF) [file pone.0158614.s005.pdf]

**S1 Table: RT-qPCR, 3' RACE and cloning primers unique to this study.**

**RT-qPCR experimental genes**

| *primer ID* | *direction* | *sequence* | *anneal* | *amplicon* | *efficiency* | *coeff. corr. ($r^2$)* | *description* |
|---|---|---|---|---|---|---|---|
| por1_fw | forward | 5' GAGGTCTCGTCTACCCACAGG 3' | 60°C | 164bp | 100.4-105.9% | 0.993-0.996 | *por*1 |
| por1_rv | reverse | 5' AAGACGGTTAAGCTCGGACAC 3' | | | | | |
| por2_fw | forward | 5' ACAATCACGAACAGACCATGC 3' | 60°C | 169bp | 100.8-103.7% | 0.994-0.995 | *por*2 |
| por2_rv | reverse | 5' AGATCGGCAATAGGGTACACG 3' | | | | | |

**3' RACE first strand synthesis**

| *primer ID* | *direction* | *sequence* | *anneal* | *description* |
|---|---|---|---|---|
| por1_out | forward | 5' TCGGATTTAGTCATGATGCG 3' | 56°C | outer nested primer |
| por1_in | forward | 5' CGTTTCCTAGTGATTTTGCTG 3' | 56°C | inner nested primer |
| por2_out | forward | 5' TACTCACCGTGTGCTTGTCA 3' | 56°C | outer nested primer |
| por2_in | forward | 5' CAAGCACCGGGGCTTTCCA 3' | 56°C | inner nested primer |

**pET-15-HE amplicon preparation for cloning**

| *primer ID* | *direction* | *sequence* | *anneal* | *description* |
|---|---|---|---|---|
| por1_he_fw | forward | 5' CTGGTGCCGCGCGGCAGCTCCATGCCCAAGGTATTGGGAGGT 3' | 53-59°C | *por*1 amplicon |
| por1_he_rv | reverse | 5' GCCGGATCCTCGAGCTAGCGCTACTATTCCTCTTCAAGCTTTCCACC 3' | | |
| por2_he_fw | forward | 5' CTGGTGCCGCGCGGCAGCTCCATGCCGGTTCATGATCCTTAT 3' | 53-59°C | *por*2 amplicon |
| por2_he_rv | reverse | 5' GCCGGATCCTCGAGCTAGCGCTACTATTCAAACTTGCTGCTCAACTT 3' | | |
